# Chromatin architecture reorganization in murine somatic cell nuclear transfer embryos

**DOI:** 10.1038/s41467-020-15607-z

**Published:** 2020-04-14

**Authors:** Mo Chen, Qianshu Zhu, Chong Li, Xiaochen Kou, Yanhong Zhao, Yanhe Li, Ruimin Xu, Lei Yang, Lingyue Yang, Liang Gu, Hong Wang, Xiaoyu Liu, Cizhong Jiang, Shaorong Gao

**Affiliations:** 1 Clinical and Translational Research Center of Shanghai First Maternity and Infant Hospital, Shanghai Key Laboratory of Signaling and Disease Research, Frontier Science Center for Stem Cell Research, School of Life Sciences and Technology, Tongji University, Shanghai, 200092 China; 2 Institute of Translational Research, Tongji Hospital, School of Life Sciences and Technology, Tongji University, Shanghai, 200065 China; 3 Institute for Regenerative Medicine, Shanghai East Hospital, Shanghai Key Laboratory of Signaling and Disease Research, Frontier Science Center for Stem Cell Research, School of Life Sciences and Technology, Tongji University, Shanghai, 200092 China; 4 Institute of Biophysics, Chinese Academy of Sciences, Beijing, 100101 China; 5 College of Life Sciences, University of Chinese Academy of Sciences, Beijing, 100049 China

**Keywords:** Embryology, Reprogramming

## Abstract

The oocyte cytoplasm can reprogram the somatic cell nucleus into a totipotent state, but with low efficiency. The spatiotemporal chromatin organization of somatic cell nuclear transfer (SCNT) embryos remains elusive. Here, we examine higher order chromatin structures of mouse SCNT embryos using a low-input Hi-C method. We find that donor cell chromatin transforms to the metaphase state rapidly after SCNT along with the dissolution of typical 3D chromatin structure. Intriguingly, the genome undergoes a mitotic metaphase-like to meiosis metaphase II-like transition following activation. Subsequently, weak chromatin compartments and topologically associating domains (TADs) emerge following metaphase exit. TADs are further removed until the 2-cell stage before being progressively reestablished. Obvious defects including stronger TAD boundaries, aberrant super-enhancer and promoter interactions are found in SCNT embryos. These defects are partially caused by inherited H3K9me3, and can be rescued by *Kdm4d* overexpression. These observations provide insight into chromatin architecture reorganization during SCNT embryo development.

## Abstract (web summary)

The organisation of chromatin in somatic cell nuclear transfer (SCNT) embryos remains poorly understood. Here, the authors examine higher order chromatin structures of mouse SCNT embryos and provide insights into chromatin architecture reorganisation during SCNT embryo development.

## Introduction

It is well recognized that somatic cell nuclear transfer (SCNT) provides the only way to reprogram somatic cells into totipotent embryos and generate viable animals^1–3^. Although various cloned animals, including amphibians^4^ and mammalians^5,6^, can be generated, the efficiency of SCNT-mediated cloning remains extremely low. Treatment with histone deacetylase inhibitors^7–9^, the overexpression of H3K9me3 demethylase *Kdm4b/4d*^10,11^, the correction of abnormal DNA re-methylation^12^ or the deletion of *Xist* on the active X chromosome^13^ can significantly improve the developmental potential of SCNT embryos, suggesting aberrant epigenetic modifications as major barriers that prevent successful reprogramming in SCNT.

Chromatin 3D structure is highly dynamic and is associated with many biological processes. Hierarchical principles of interphase chromatin 3D structure include chromosome territories, chromatin compartments(A/B), TADs and loops. A and B compartments are two kinds of multi-megabase domains characterized by the spatial segregation of active and inactive chromatin^14^. Extensive A/B compartments switching during stem cell differentiation indicates that they are cell type-specific^15^. TAD is identified as contiguous chromatin region that contains loci with high-frequency interactions inside it, and contacts between TADs are insulated^16^. Although most TADs are relatively conserved during cell differentiation, the interaction frequency within some domains is different between cell types^15^. Therefore, proper 3D chromatin structure establishment is an important step during cell fate transition.

With low-input in situ Hi-C techniques, the drastic dynamics of chromatin organization in early embryo development can be detected^17–20^. In *Drosophila* embryos, higher order chromatin structure emerges during zygotic genome activation (ZGA) and TAD boundary formation is transcription independent^19^. In zebrafish embryos, chromatin structure undergoes a process of loss and rebuilding^20^. In mouse embryos, higher order chromatin architecture gradually matures during development which is transcription independent^17,18^. However, little is known about the reprogramming of 3D chromatin structure during SCNT embryo development.

Here, we examine the 3D chromatin structure across consecutive stages of SCNT embryo development and find that higher order chromatin architectures, including compartments and TADs, are dissolved and reestablished in a stage-specific and coordinated manner during SCNT embryogenesis. H3K9me3 modification is likely an epigenetic barrier that impairs the reprogramming of chromatin architecture during SCNT embryo development. Therefore, our findings provide a high-resolution map of how the mature 3D chromatin structure of somatic cells is reprogrammed to a totipotent state after transplanting into enucleated oocytes.

## Results

### The 3D chromatin structure of SCNT embryos

**Fig. 1:**
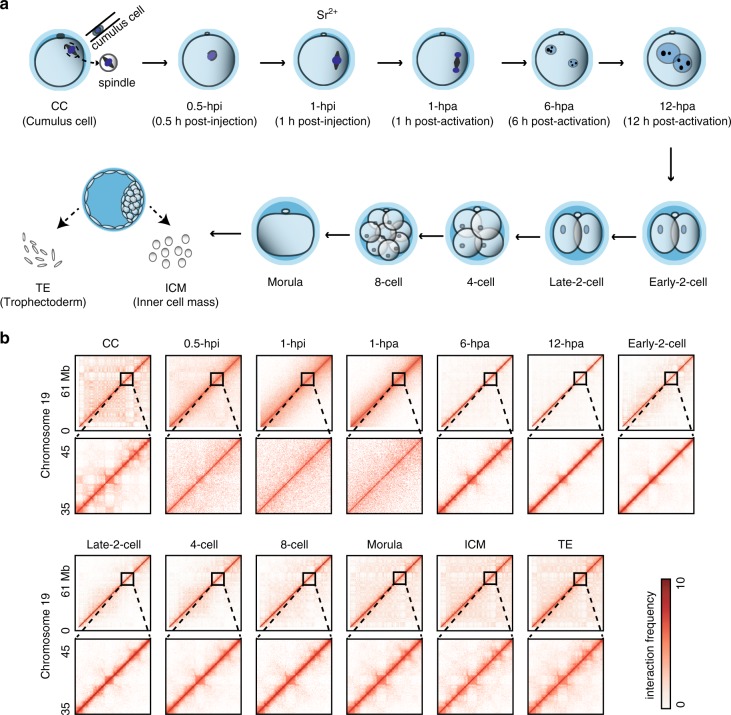
**Landscape of chromatin organization in early SCNT embryo development**. **a** Illustration of SCNT procedure and sample collections. According to the classical procedure of mouse cloning, donor nuclei from cumulus cells were injected into enucleated oocytes. Reconstructed embryos were chemically activated 1 h post-injection by culture in the Ca^2+^-free CZB medium including strontium chloride. **b** Hi-C produces a genome-wide contact matrix. The normalized Hi-C interaction frequencies (100-kb bin, chromosome 19) in each sample. Zoomed-in views (40-kb bin) are also shown. Each pixel represents all interactions between a 100-kb (40-kb) locus and another 100-kb (40-kb) locus; intensity corresponds to the ICE normalized value (0–10).

Extensive chromatin architecture reorganization, which is critical for gene expression, occurs during preimplantation embryo development in mammals. To reveal the establishment of higher order chromatin structure during the early development of SCNT embryos, we optimized a small-scale in situ Hi-C (sisHi-C) method based on a recent study^17^. We generated high-quality Hi-C data using 100–500 mouse ES cells that were accurately consistent with previously reported chromatin interaction patterns and architecture (Supplementary Fig. 1a–d). We next collected mouse cumulus cells (CCs), which were used as donor cells for SCNT, and reconstructed embryos at different stages, including the 0.5 h post-injection (0.5-hpi), 1-hpi, 1 h postactivation (1-hpa), 6-hpa, 12-hpa, early-2-cell embryo, late-2-cell embryo, 4-cell embryo, 8-cell embryo, morula embryo, as well as inner cell mass (ICM) and trophectoderm (TE) from blastocyst stage embryos and performed Hi-C experiments at each stage (Fig. 1a, Supplementary Table 1). The Hi-C data of replicates were highly reproducible (Supplementary Fig. 1e). Consistent with the reported features associated with higher order chromatin architecture^14,16,18^, active histone modification H3 lysine 4 trimethylation (H3K4me3) was primarily enriched in compartment A, whereas repressive H3 lysine 27 trimethylation (H3K27me3) was primarily enriched in compartment B (Supplementary Fig. 1f–h). Similarly, H3K4me3 was enriched at the boundaries of TADs, whereas H3K27me3 was depleted (Supplementary Fig. 1i). Additionally, short interspersed element (SINE) retrotransposons, CpG islands and gene promoters were also enriched in TAD boundaries (Supplementary Fig. 1j).

### Metaphase transition following SCNT embryo activation

The examination of chromatin organization at each stage showed that typical higher order chromatin architectures, for example, TADs and compartments in the terminally differentiated CCs, were rapidly dissolved after the injection of CC nuclei into enucleated oocytes and were partially recovered in 6-hpa embryos (Fig. 1b). Strikingly, 1-hpi and 1-hpa one-cell embryos had significantly fewer distal interactions and showed a uniform interaction pattern (Fig. 1b), which was highly similar to that observed in metaphase chromatin organization^21–23^.

**Fig. 2:**
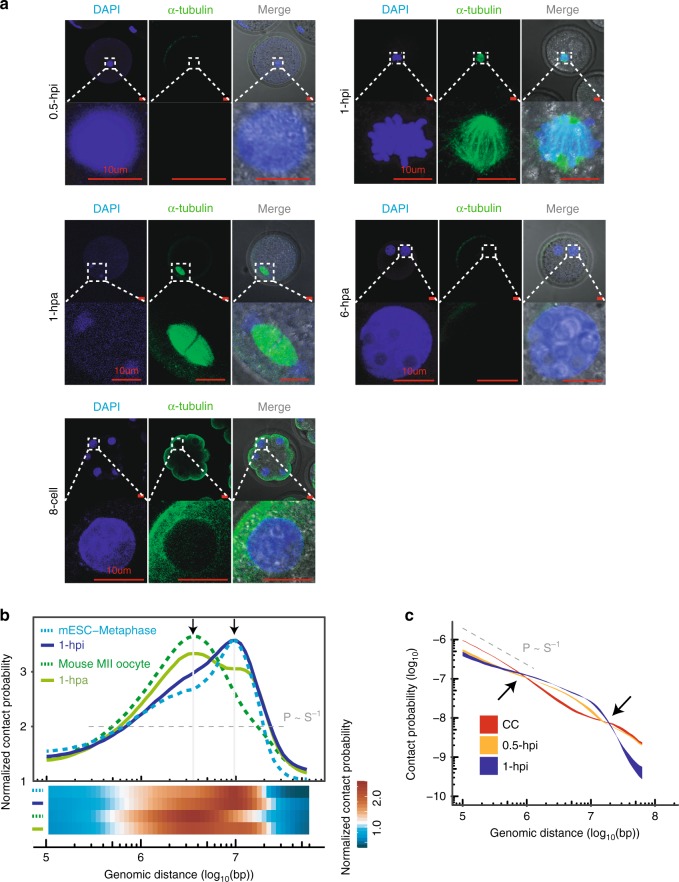
**Mitotic metaphase to meiosis metaphase II-like chromatin transition**. **a** Immunofluorescence staining of 0.5-hpi, 1-hpi, 1-hpa and 6-hpa 1-cell embryos and 8-cell embryos. DNA: DAPI (blue) and microtubules: α-tubulin (green). Scale bar: 10 μm. Data are representative of three independent experiments. **b** Both the curves and heatmaps showing the intrachromosomal interaction probabilities (*P(s)*) relative to genomic distance that are normalized by the *P(s) ~ s*^−1^ values. The peaks represent the rapid fall-off at ~4 Mb and ~10 Mb in secondary meiotic and mitotic chromatin, respectively. Source data are provided as a Source Data file. **c** The chromatin contact probabilities (*P(s)*) relative to genomic distance for CC, 0.5-hpi, and 1-hpi embryos. The *P(s) ~ s*^−1^ curve representing the predicted fractal globule state is shown for reference. The *P(s)* ribbon is bounded by minimum and maximum (*P(s)*) calculated from all replicates of Hi-C data sets. Source data are provided as a Source Data file.

We then analyzed chromatin conformation at the 1-hpi and 1-hpa stages. Consistently, the *P(s)* curves (chromatin contact probabilities relative to the genomic distance) for 1-hpi and 1-hpa embryos better matched the *P(s) ~ s*^−0.5^ curve, representing the predicted metaphase chromatin state; the *P(s)* curves for CCs, ICM and TE better matched the *P(s) ~ s*^−1^ curve, representing the predicted fractal globule state (interphase chromatin state)^14^ (Supplementary Fig. 2a). Immunofluorescence staining detected condensed chromosomes with a spindle apparatus in 1-hpi and 1-hpa embryos, which was distinguishable from interphase chromatin morphology with a nuclear envelope in other-stage embryos (Fig. 2a). This result was also consistent with a previous study showing that donor cells underwent nuclear envelope breakdown and premature chromosome condensation after fusion with oocytes^24,25^. Moreover, the metaphase-like chromatin state was characterized by a lack of interchromatin interactions^21^, which is consistent with the chromatin structure at 1-hpi and 1-hpa stage embryos (Supplementary Fig. 2b). This result indicated that the somatic cell genome was converted to a metaphase-like state shortly after injection into the oocyte cytoplasm (at the 1-hpi and 1-hpa one-cell stages).

Further analysis demonstrated that the *P(s)* curve for 1-hpi embryos resembled that of the mouse ESC metaphase with a rapid decline at 10 Mb. In contrast, the *P(s)* curve for 1-hpa embryos resembled that of mouse MII oocytes with an abrupt decrease until 4 Mb (Fig. 2b; Supplementary Fig. 2c). This result was consistent with previous findings that chromatin interactions were precipitously reduced beyond 4 Mb in MII oocytes^17^ and beyond 10 Mb in mitotic chromatin^17,21,22^, suggesting that the mitotic metaphase-like chromatin state in 1-hpi embryos transformed into the meiosis metaphase II-like chromatin state after activation.

### Short-distance interaction dissolves first after SCNT

Accurate spatiotemporal chromatin packaging is critical to embryogenesis^19,20^. Thus, we compared the *P(s)* curves between consecutive stages. After injection of CC nuclei into enucleated oocytes, short-distance (<1 Mb) interactions decreased, and intermediate-distance (1–10 Mb) interactions increased within the first 30 min. However, long-distance (>10 Mb) interactions required a longer time to change, and are significantly decreased at the 1-hpi stage (Fig. 2c, Supplementary Fig. 2d). This result indicated that reorganization from the interphase to metaphase-like chromatin structure started from short-distance interactions and progressed to long-distance interactions. These findings are consistent with a recent study, which showed that long-range compartmentalization occurs more slowly during mitotic exit and G1 re-entry^26^. Intriguingly, spatiotemporal chromatin packaging was reversed at the 6-hpa stage with increased short-distance interactions and decreased intermediate-distance interactions. The short-distance interactions changed very little after 6-hpa and decreased to the level of that in the ICM stage. In contrast, intermediate- and long-distance interactions increased at the early-2-cell stage, and extra long-distance (>20 Mb) interactions increased in both the morula and ICM stages (Supplementary Fig. 2e). Collectively, these results suggest that spatiotemporal chromatin packaging is a coordinated process involving higher order dynamics in early stages and ultimately chromatin compaction in the ICM stage.

### Reprogramming of compartments during SCNT embryo development

**Fig. 3:**
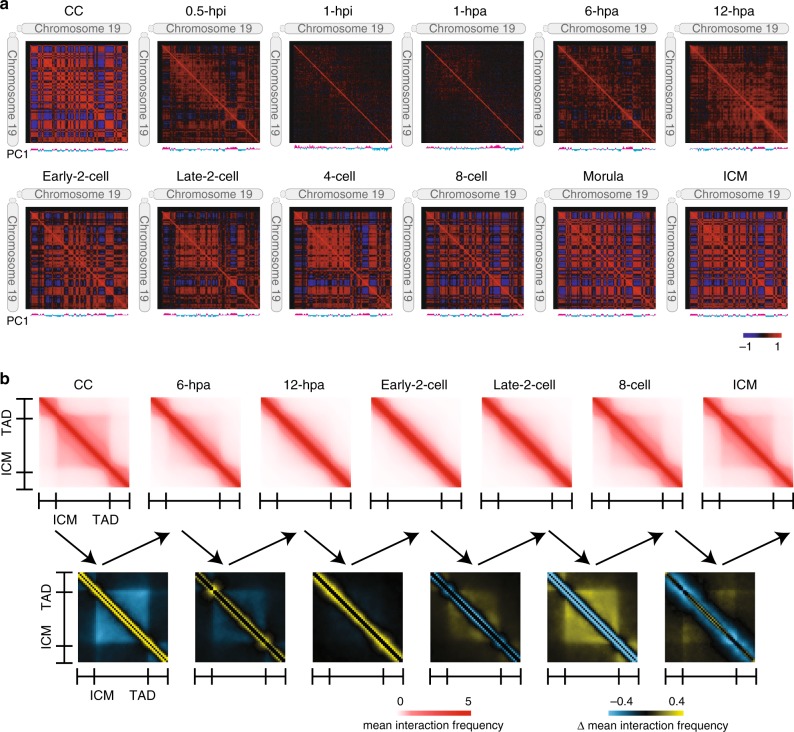
**Dissolution and reestablishment of 3D chromatin architecture**. **a** Correlations of intrachromosomal interaction frequency patterns between any two regions along chromosome 19 (300-kb bin). The first principle component (PC1) values are shown under each heatmap as compartments A (magenta) and B (cyan). **b** The top heatmaps show the mean normalized interaction frequencies for the TADs and their flanking regions with a TAD length of ±0.5. The TADs were defined in the ICM stage and used for all stages. The bottom heatmaps show the difference in the interaction frequencies between consecutive stages. Source data are provided as a Source Data file.

Compartments are typical higher order chromatin structures that contribute to determining cell type-specific patterns of gene expression^15,27^. We observed clear plaid patterns of chromatin interactions in the correlation heatmap across all stages except for the 1-hpi and 1-hpa stages (Fig. 3a). These plaid patterns represent chromatin compartments A and B^14^. To understand the reprogramming of chromatin compartments during SCNT embryo development, we first identified compartments across all stages (see Methods). Consistent with the metaphase-like chromatin state, compartments were disassembled in 1-hpi and 1-hpa embryos and had no correlation with mature compartments in CCs or the SCNT ICM (Supplementary Fig. 3a, b). This result indicated that the mature compartments in CCs were substantially dissolved after injection into enucleated oocytes, leading to poor chromatin compartmentalization in 1-hpi and 1-hpa embryos. Nevertheless, the compartments were consistent for the other stages. We then analyzed compartmental switching during SCNT embryo development and collected two groups of compartments. One group consisted of 752 compartments A in CCs that gradually became compartments B in the ICM. In contrast, the other group of 1046 compartments B in CCs gradually became compartments A in the ICM (Supplementary Fig. 3c). The expression levels of genes in the A to B group of compartments, which included the CC-specific gene *Has2*, significantly decreased along with the compartmental switch progressed^28^. The expression levels of genes in the B to A group, which included the placenta-associated gene *Pramel4*, exhibited the opposite trend^29^ (Supplementary Fig. 3d–h).

### Reprogramming of TADs during SCNT embryo development

TADs are another typical higher order chromatin structure that have “self-association” and “insulation” properties^16,30,31^. To investigate how the TADs in CCs were dissolved and reestablished during SCNT embryo development, we identified 2516 TAD boundaries in CCs and 1941 TAD boundaries in the ICM using the insulation score as previously described^32^. Hi-C interaction heatmaps showed that both the distal (>2 Mb) chromatin interactions and TADs in CCs were rapidly dissolved after injection into the enucleated oocyte. Chromatin interactions were locus-independent and uniform at the 1-hpi and 1-hpa stages, and TADs were also completely dissolved (Supplementary Fig. 4a–b). These results depicted the characteristic pattern of metaphase chromatin interactions^21,22^. Both TADs and sparse distal interactions emerged at the 6-hpa stage. The emergence of high-order structures was due to exiting the metaphase-like chromatin state because dissolved TADs could gradually regenerate after the metaphase stage was complete^21,22^. The newly emerged TADs at the 6-hpa stage were weaker than those in CCs, and continue to weaken until the early-2-cell stage followed by reestablishment. Once TADs were established, they were consistent in later stages (Fig. 3b, Supplementary Fig. 4a–f). These data indicated the removal and reestablishment of TADs during SCNT embryo development.

### Aberrant chromatin structure organization of SCNT embryos

**Fig. 4:**
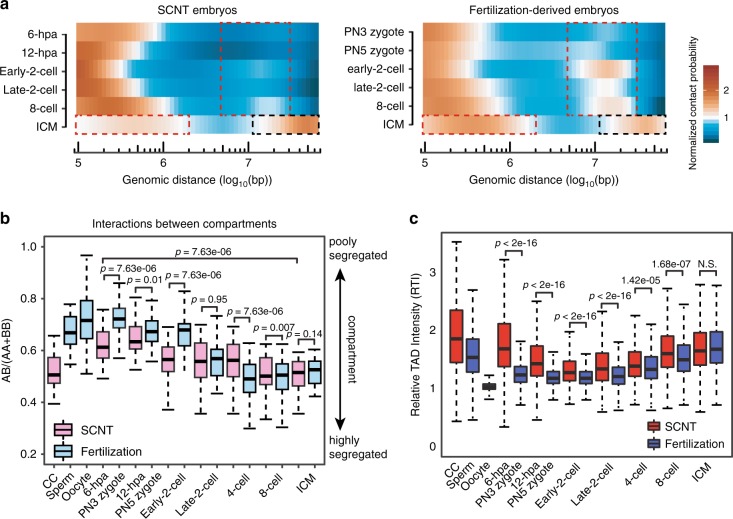
**Differential dynamic of chromatin structure reorganization between normal and SCNT embryos**. **a** Heatmaps showing the intrachromosomal interaction probabilities relative to genomic distance that are normalized by the *P(s) ~ s*^−1^ values. Dashed boxes indicate the developmental stages and genomic distance with differential interaction probabilities between SCNT and fertilization-derived embryos. Source data are provided as a Source Data file. **b** Ratios of interaction frequencies between compartments A & B to those between compartments A & A or B & B (*n* = 323). Boxes show 25th, 50th and 75th percentiles and whiskers show 1.5× the inter-quartile range. The p values were calculated by a two-sided Wilcoxon rank-sum test with BH multiple testing correction Source data and exact p-value are provided as a Source Data file. **c** RTI across all stages (*n* = 2057). Boxes show 25th, 50th and 75th percentiles and whiskers show 1.5× the inter-quartile range. The two-sided *p* values were calculated by the Kruskal–Wallis test with Dunn’s multiple comparison test and adjusted by default with the holm method (N.S., not significant). Source data and exact p-value are provided as a Source Data file.

It is intriguing to compare the different chromatin structure reprogramming patterns between SCNT and fertilization-derived embryos, which should contribute to their differential embryo developmental potential. We first compared the interaction heatmaps between SCNT and fertilization in early 2-cell embryos. There is more distinct interdomain regional separation in SCNT embryos than in fertilization-derived embryos. In addition, distal (>2 Mb) interactions are also weaker in SCNT embryos (Supplementary Fig. 5a). Then we compared the *P(s)* curves for SCNT and fertilization-derived early-2-cell embryos from all independent replicates of Hi-C datasets. Consistently, the distal contact probabilities were higher in fertilization-derived embryos. Note that there was a turning point at ~10 Mb distance that maximized the difference in contact probability between fertilization-derived and SCNT embryos (Supplementary Fig. 5b). To preclude the distance effect, we further normalized the relative contact probability to the reference curve *P(s) ~ s*^−1^. We found that the frequencies of distal (>2 Mb) interactions in the early stages of SCNT embryo development (6-hpa and 12-hpa) were lower than those in fertilization-derived embryos (PN3 and PN5). This difference became obvious at the early-2-cell, late-2-cell and 8-cell stages. At the ICM stage, extra long-distance (>20 Mb) interactions were more abundant in the SCNT embryos than in the fertilization derived embryos, while proximal interactions exhibited the opposite trend (Fig. 4a). This differential spatiotemporal chromatin packaging was confirmed by the number of interaction read pairs as well (Supplementary Fig. 5c, d).

We next compared chromatin compartment interactions between SCNT and fertilization-derived embryos. Although compartments A & B became more segregated in both SCNT and fertilization-derived embryos during development, compartments A & B were more poorly segregated in fertilization-derived embryos until the early-2-cell stage than in SCNT embryos (Fig. 4b), which is likely attributed to the substantially fewer intercompartmental interactions occurring in CCs than in sperm and oocytes. We then clustered regions based on the first component (PC1) values across embryo developmental stages and identified a set of regions that were active in compartment A in early stages (PN5, 2-cell) of fertilization-derived embryo development but repressive in compartment B in early stages of SCNT embryo development (Supplementary Fig. 5e). A previous study found a group of genes that were not activated in SCNT 2-cell-arrest embryos, including *Foxa2*^10^. We also found that the compartmental state of *Foxa2* was not reprogrammed to compartment A at the 2-cell stage in SCNT embryos (Supplementary Fig. 5f). As a result, the ZGA process may be defective during SCNT embryo development.

Next, we examined differences in TADs between fertilization-derived and SCNT embryos. The general trend was that TADs became mature in the ICM during both fertilization-derived and SCNT embryo development (Fig. 4c). However, the TAD structure was stronger in SCNT embryos than in fertilization-derived embryos until the 8-cell stage. The TAD intensity difference was gradually attenuated during embryo development. The difference in TADs was also likely attributed to the stronger TADs in CCs than in sperm and oocytes. Notably, there are still many distinct TADs between the SCNT and fertilization-derived ICMs (Supplementary Fig. 5g). Taken together, these results suggest that the high-order chromatin structures of SCNT and fertilization-derived embryos differ substantially in the early stages, such as the 2-cell stage, and become increasingly similar during embryo development.

### Absence of SE–P interaction of ZGA genes in SCNT embryos

High-order chromatin structure has important roles in gene regulation^33,34^. Genes could be modulated by regulatory elements located in the same TAD, such as typical enhancers. In addition, super-enhancers (SEs), a large domain clustered by typical enhancers, bind with high-density of transcription factors and coactivators and regulate gene expression by forming long-distance interactions^35^. Therefore, we speculated that such differential chromatin packaging impacted interactions between super-enhancers and promoters. To validate this hypothesis, we first identified super-enhancers as described previously^35^ (see Methods for details). We next grouped chromatin interactions between super-enhancers and promoters as SE–P interactions and the remaining interactions as nonSE–P interactions using Fit-HiC tools^36^. More than 90% of SE–P interactions occurred in compartment A during both SCNT and fertilization-derived embryo development, whereas approximately 50% of the nonSE–P interactions occurred in compartment B (Supplementary Fig. 6a). In addition, target genes associated with SE–P interactions had significantly higher expression levels than genes associated with nonSE–P interactions, such as promoter–promoter (P–P) interactions (Supplementary Fig. 6b). These results suggested that the SEs identified in this study were reliable.

Further analysis demonstrated that the overlap of SE–P interactions between fertilization-derived and SCNT embryos increased during development. (Supplementary Fig. 6c). This suggested that the higher order chromatin structure of SCNT embryos becomes more similar to that of fertilization-derived embryos during development. We then identified all genes that were regulated by SE–P loops and activated in the fertilization-derived ICM but lacked SE–P loops and were repressed in the SCNT ICM. Gene Ontology analysis of these genes identified the enrichment of fundamental biological processes, such as protein transport, DNA-templated transcription, cellular response to DNA damage stimulus, and cell–cell adhesion (Supplementary Fig. 6d). This result implied that the aberrant SE–P interactions resulting from differential chromatin packaging during SCNT embryo development extensively impacted biological processes and likely contributed to the low SCNT embryo development rate.

**Fig. 5:**
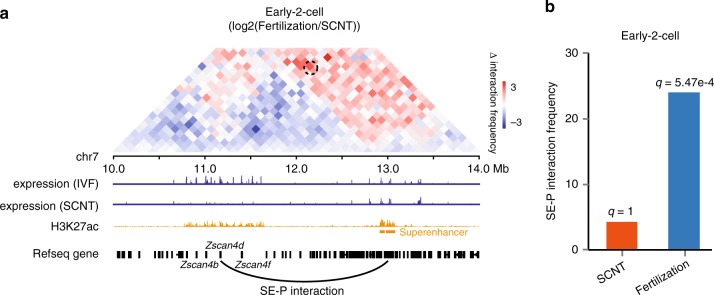
**Defects of promoter and super-enhancer interactions in SCNT embryos**. **a** The top heatmap shows the differential interaction frequencies at the *Zscan4d* locus between fertilization-derived and SCNT early-2-cell embryos. The dashed circle indicates the SE–P interactions in fertilization-derived embryos but not in SCNT embryos. The bottom tracks show gene expression in IVF and SCNT embryos, H3K27ac signals in fertilization-derived 2-cell embryos, and Refseq genes, respectively. Source data and exact p-value are provided as a Source Data file. **b** The normalized interaction frequencies between the super-enhancer and promoter of *Zscan4d*. The *q*-values were calculated in Fit-Hi-C by applying Benjamini-Hochberg correction to the *p*-values, as computed by the binomial distribution model employed.

ZGA is a critical event during early embryonic development. To investigate the relationship between chromatin organization and ZGA, we focused our analysis on differential SE–P interactions at the early-2-cell stage and found that SE–P interaction at the *Zscan4d* (~2 Mb) locus was obvious in fertilization-derived 2-cell embryos but absent in SCNT early-2-cell embryos (Fig. 5a, b; Supplementary Fig. 6e). We further used FIND tools^37^ to analyze the differential chromatin interactions around these loci and verified this difference(Supplementary Fig. 6f). The *Zscan4d* gene is specifically expressed at the 2-cell stage and is essential for preimplantation development^38^, but it is silenced in SCNT 2-cell embryos^11^. This result implied that the aberrant SE–P interactions during SCNT embryo development may be responsible for the abnormal gene activation and the low embryo developmental rate.

### H3K9me3 is a barrier for chromatin structure reprogramming

**Fig. 6:**
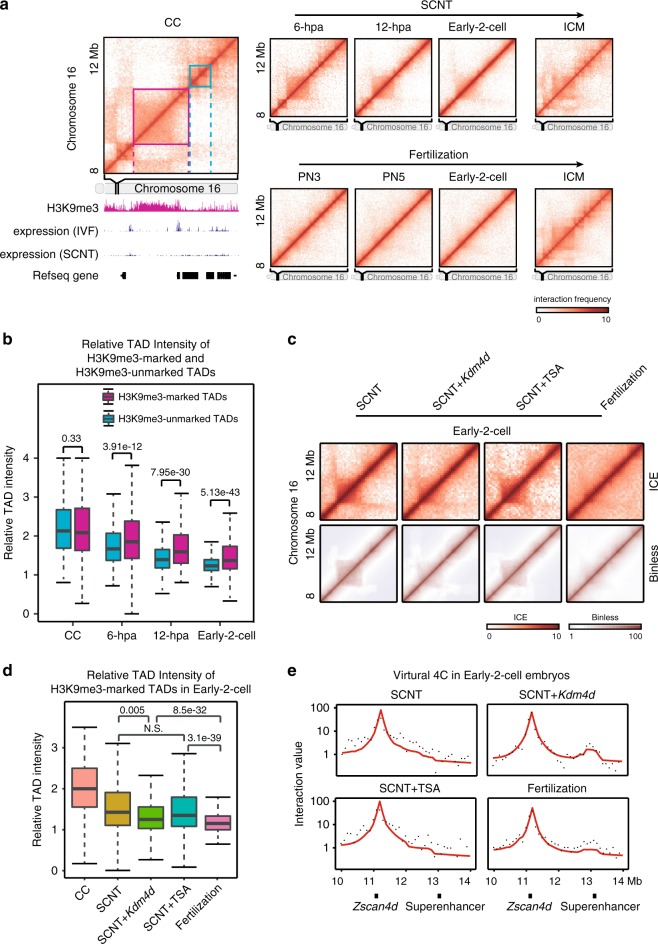
**H3K9me3 is a potential barrier of TADs reprogramming**. **a** Heatmaps of chromatin interaction frequencies showing a CC TAD unreprogrammed example (magenta) and a CC TAD reprogrammed (cyan) example during SCNT embryo development. The bottom track shows the H3K9me3 signal in CCs. **b** RTIs of H3K9me3-maked and H3K9me3-unmarked TADs identified in CCs. RTI values in CC, 6-hpa, 12-hpa and early-2-cell embryos are shown (*n* = 2184). Boxes show 25th, 50th and 75th percentiles and whiskers show 1.5× the inter-quartile range. The two-sided p values were calculated by the Kruskal–Wallis test with Dunn’s multiple comparison test and adjusted by default with the holm method. Source data and exact p-value are provided as a Source Data file. **c** Heatmaps showing normalized chromatin interaction frequencies (100-kb bin, chromosome 16) in SCNT, SCNT with *Kdm4d* mRNA injection, SCNT with TSA treatment and fertilization-derived early 2-cell embryos. Both ICE and Binless normalization approaches are used. **d** RTIs of H3K9me3-maked TAD in early 2-cell embryos. RTI values in CC, SCNT, SCNT with *Kdm4d* mRNA injection, SCNT with TSA treatment and fertilization-derived are shown (*n* = 2184). Boxes show 25th, 50th and 75th percentiles and whiskers show 1.5× the inter-quartile range. The two-sided *p* values were calculated by the Kruskal–Wallis test with Dunn’s multiple comparison test and adjusted by default with the holm method (N.S., not significant). Source data and exact p-value are provided as a Source Data file. **e** Virtual 4C test showing the interaction frequencies between *Zscan4d* promoter and its adjacent region in chromosome 7. Black dots represent observed values, red line represents fitted value by Binless.

Finally, we attempted to explore the possible mechanisms underlying the differential reprogramming of chromatin organization during SCNT embryo development. A previous study identified three types of regions in SCNT embryos: RRRs (reprogramming-resistant regions), PRRs (partially reprogrammed regions) and FRRs (fully reprogramming regions)^11^. Intriguingly, we found that RRRs were significantly enriched in compartment B in SCNT embryos (Supplementary Fig. 7a). In addition, the insulation score was significantly higher in RRRs than in PRRs and FRRs (Supplementary Fig. 7b). More RRRs are located far from the boundary position than PRRs or FRRs in SCNT and fertilization-derived embryos (Supplementary Fig. 7c). These results suggested that RRRs more likely resided within TADs than boundaries. As H3K9me3 enrichment in the donor cell genome contributes to the reprogramming defects of RRRs^11^, and TAD boundaries have the potential to restrict the spread of repressive chromatin into active domains^39^, we hypothesized that the TAD structure associated with H3K9me3 may exhibit high-order chromatin structure reprogramming defects during SCNT embryo development. To validate this hypothesis, we first identified H3K9me3-marked and H3K9me3-unmarked TADs in CCs (see Methods). RRRs were more enriched in H3K9me3-marked TADs than in FRRs and PRRs (Supplementary Fig. 7d). We also found that the H3K9me3-marked CC TADs failed to disassemble in SCNT embryos, though no TADs were observed in the corresponding regions of fertilization-derived embryos. In contrast, H3K9me3-unmarked CC TADs were rapidly dissolved in SCNT embryos (Fig. 6a). Although both types of TADs had similar relative TAD intensities (RTI) in CCs, the intensity remained significantly higher in H3K9me3-marked TADs than in H3K9me3-unmarked TADs after SCNT (Fig. 6b). We further compared the RTI values of TADs between SCNT 12-hpa embryos and in vitro fertilization (IVF) PN5 zygotes and defined two types of TADs: reprogrammed TADs and unreprogrammed TADs (see Methods). H3K9me3 signals in the CC genome were significantly higher in unreprogrammed TADs than in reprogrammed TADs (Supplementary Fig. 7e). Finally, we clustered TADs in the fertilization-derived ICM that were in a “mature” state based on RTI values across developmental stages of SCNT and fertilization-derived embryos. A cluster of TADs (C2) had strong RTI values in SCNT 2-cell embryos but low RTI values in IVF 2-cell embryos (Supplementary Fig. 7f). Therefore, these TADs remained largely unreprogrammed in SCNT 2-cell embryos. Coincidently, these TADs (C2) had significantly higher H3K9me3 signals in the CC genome than the other clusters of TADs (C3–C5) that were well reprogrammed in SCNT embryos (Supplementary Fig. 7g).

To verify the effect of H3K9me3 retention on TAD reprogramming, we injected *Kdm4d* mRNA, H3K9me3 demethylase, into the SCNT embryos and the histone deacetylase inhibitors, Trichostatin A (TSA), treatment was also performed as a control. Interaction heatmaps show weaker TAD signals in the SCNT-*Kdm4d* group than in the SCNT or SCNT-TSA group (Fig. 6c). Box plot shows significantly decreased RTI values in H3K9me3-marked TADs in the SCNT-*Kdm4d* group compared with those in the SCNT and SCNT-TSA groups, but these values were still higher than those in fertilization-derived embryos (Fig. 6d). On the other hand, we found that the SE–P interaction at the *Zscan4d* locus was lost in SCNT 2-cell embryos and was recovered in SCNT-*Kdm4d* embryos (Fig. 6e; Supplementary Fig. 7 h). This is probably due to the relatively unconsolidated chromatin boundary structure between them after *Kdm4d* overexpression. This result implied that H3K9me3 enrichment in the CC genome was an epigenetic barrier and impeded the SCNT-mediated reprogramming of chromatin architecture, and the injection of *Kdm4d* mRNA could partially rescue un-disassembled H3K9me3-marked TADs in SCNT early-2-cell embryos.

## Discussion

**Fig. 7:**
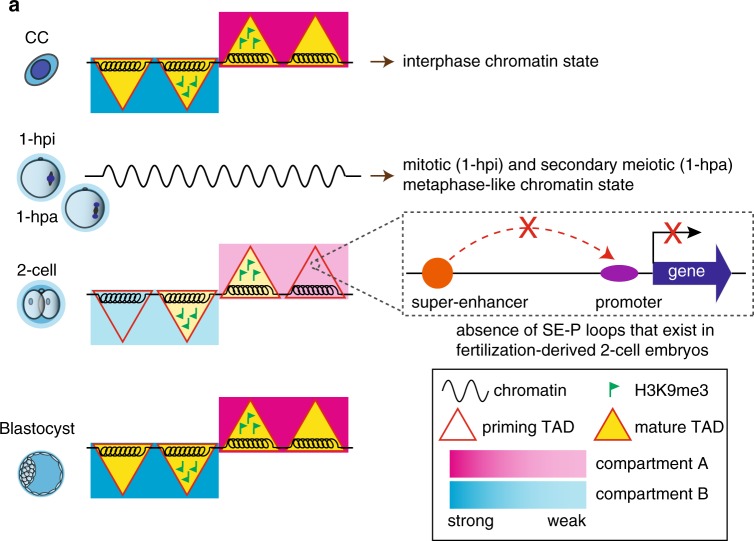
**A schematic model showing the 3D chromatin structure dynamic during SCNT embryo development**. **a** CCs exhibit interphase-state chromatin characterized by mature compartments and TADs, both of which are dissolved quickly after injection into enucleated oocytes. Consequently, 1-hpi and 1-hpa embryos exhibit a mitotic and secondary meiotic metaphase-like chromatin state lacking compartments and TADs, respectively. Compartments and TADs emerge in early-2-cell embryos, exist in later stages, and become mature in the ICM stage. TADs with enriched H3K9me3 signals are resistant to reprogramming. The differential reprogramming of chromatin architecture results in the loss of SE–P loops of  regulating genes critical to development.

Although the reprogramming of higher order chromatin architecture during the early development of mouse fertilization-derived embryos has been studied^17,18^, it remains largely unexplored during SCNT embryo development. Therefore, the difference in 3D chromatin reorganization between fertilization-derived and SCNT embryos is completely unknown. The molecular basis underlying differential chromatin reorganization also remains unclear. Here, we examined the 3D chromatin structures across consecutive stages of SCNT embryo development and found that the general profiles of chromatin architecture reprogramming were similar between fertilization-derived and SCNT embryos. The interphase chromatin state in CCs was reorganized into a metaphase-like state shortly after injection into the enucleated oocyte. Higher order chromatin architectures, including compartments and TADs, were dissolved. TADs with weak boundary insulation emerged as early as the 6-hpa stage through recovery from exiting the metaphase-like state. Taken together, these results suggest that higher order chromatin architecture is dissolved and reestablished in a stage-specific and coordinated manner during SCNT embryogenesis. Intriguingly, many SE–P interactions critical for embryo development are lost during SCNT embryo development. The enrichment of H3K9me3 modifications in the donor cell genome is likely an epigenetic barrier that impairs the reprogramming of chromatin architecture during SCNT embryo development (Fig. 7a).

SCNT efficiency is very low in terms of blastocyst development and the birth of full-term animals^40,41^. Our work suggests that higher order chromatin structure can be rapidly reorganized to an embryo like state during SCNT-mediated reprogramming. However, many abnormalities in cloned embryos have been observed, which may be responsible for the low developmental rate of SCNT embryos. Promoter-enhancer interactions are highly dynamic during SCNT reprogramming, as reported in lineage specification^15^. The failed reprogrammed SE–P interactions may result in abnormalities in gene expression during ZGA in SCNT embryos. Additionally, the TAD structure was stronger in SCNT embryos than in fertilization-derived embryos, which may be linked to the decreased long-range interaction in SCNT embryos. At the gene expression level, failure to silence donor cell-specific genes may lead to an aberrant embryonic phenotype in SCNT embryos^10,42,43^. The reprogramming-resistant TADs and boundaries inherited from the donor cell may be related to aberrant gene expression (Supplementary Fig. 6e). *Kdm4d* overexpression can improve the SCNT efficiency partially by weakening the TAD intensity, suggesting that the TAD boundaries in donor cells are also a barrier for SCNT reprogramming. Therefore, manipulating the expression level of CTCF may be helpful for improving SCNT efficiency, as partial depletion of CTCF weakens TAD boundaries and improves interdomain interactions^44^. We find the 3D chromatin structure between SCNT and fertilization-derived embryos is getting similar. But there are still many defects, such as aberrant SE–P interactions and more long-range interactions in the ICM of SCNT embryos. This indicates that the reprogramming of genome structure is not completely accomplished, even at the blastocyst stage, which can partially explain the low rate of implantation and full-term development of SCNT embryos^41^.

Chromosomal condensation is required for establishing an embryonic pattern of DNA replication and allowing cell cycle progression in SCNT embryos^45^. In frog oocytes, chromosome condensation is associated with the transition between different DNA replication programs^46^. In this study, we observe that the interphase type chromatin structure changes rapidly to a mitotic metaphase state following nuclear transfer, and the 3D chromatin structure is removed and reestablished following the cell cycle transition. When we shaded the metaphase stage, we find that the TADs are gradually removed from the somatic cell to the 2-cell stage embryo and then progressively reestablished until the blastocyst stage (Fig. 4c), which indicates that the removal of TADs does not merely depend on the cell cycle transition during SCNT reprogramming.

Maternal factors within the oocyte cytoplasm direct the rapid reprogramming of the chromatin structure after injection and activation. According to a previous work published by our laboratory^47^, we found that the critical reprogramming factors reside predominantly in the male pronucleus when we utilized zygotes as recipients during SCNT. Screening for factors which are particularly expressed in the male pronucleus by quantitative mass spectrometry analysis may be a valid approach to search for these key reprogramming factors. On the other hand, metaphase transition seems to be a critical step for the dissolution of 3D chromatin structure during SCNT, which indicates that cell cycle related factors might also play a role in the rapid change of chromatin structure.

SCNT is the only way for differentiated somatic cells to obtain totipotency. One of the most significant findings in our study is the metaphase-like transition from mitotic to meiosis II during SCNT embryo development, which may be a critical step to gain totipotency, because there was no such transition during iPSC reprogramming^48^. Our results also suggest that searching for oocyte cytoplasmic factors which are responsible for the transition may help us induce and maintain totipotency directly from somatic cells in vitro.

ZGA is a critical step for mammalian embryo development. Major ZGA occurs at the 2-cell stage in mice, and many ZGA genes are transiently expressed, with *Zscan4d* as a well-known example^49^. Knocking down or sustained expression of *Zscan4* impairs embryo development and implantation^38^. However, the mechanism of transient expression of ZGA genes has not yet been elucidated. In this study, we provide a possible regulatory mechanism of *Zscan4d* by SE–P interaction. Moreover, we can rescue this interaction by reducing the TAD intensity by removing H3K9me3 modification in SCNT embryos (Fig. 6e). We also find many other failed reprogrammed SE–P interactions in SCNT embryos and whether these genes are important to ZGA and embryo development deserves further investigation.

## Methods

### Animals and SCNT procedure

Specific pathogen-free grade mice were housed in the animal facility of Tongji University, Shanghai, China. All animal maintenance and experimental procedures were performed in accordance with the Tongji University Guide for the Use of Laboratory Animals.

Eight- to ten-week-old B6D2F1 (C57BL/6×DBA/2) female mice were superovulated by injection with 5 IU of pregnant mare serum gonadotropin (PMSG), followed by injection of 7 IU of human chorionic gonadotropin (hCG, San-Sheng Pharmaceutical) 48 h later. Mouse MII oocytes were retrieved from the dissected oviducts at 13 h post-hCG injection and incubated in Chatot-Ziomek-Bavister medium (CZB) at 37 °C and 5% CO_2_ until use. CCs were removed by incubating the cumulus-oocyte complex in hyaluronidase from bovine testes (0.5 μg ml^−1^, Sigma) at 37 °C for 3–5 min. Fully dissociated cumulus cells were collected and washed in Hepes-CZB and then stored at 4 °C until nuclear transfer manipulation. Mouse MII oocytes were enucleated in Hepes-CZB containing 5 μg ml^−1^ cytochalasin B (Sigma) at 37 °C. The inner diameter of the transfer needles used for CC nucleus was 5–6 μm. The somatic cell was administered 1–2 piezo pulses (intensity 1 or 2) while at the tip of the transfer needle to break the plasma membrane. Then, the cell was immediately injected into an enucleated oocyte in Hepes-CZB containing 5 μg ml^−1^ cytochalasin B. The plasma membrane of the enucleated oocyte was broken with a single piezo pulse, the cumulus cell was injected, and the membrane was sealed by aspiration of a small amount of cytoplasm. Reconstructed embryos were cultured in CZB at 37 °C and 5% CO_2_ for 1 h and activated for 5 h using strontium chloride in calcium-free CZB in the presence of 5 μg ml^−1^ cytochalasin B to prevent the extrusion of a pseudopolar body. The reconstructed embryos were then transplanted into G1 medium and cultured to the blastocyst stage.

For TSA-treated group, extra 50 nM Trichostatin A (Sigma, T8552) were added to the activation and culture mediums for 9 h after somatic cells injection.

### Sample harvest for the Hi-C experiment

Embryos at each of the following stages were collected: 0.5 h and 1 h post-injection; 1 h, 6 h and 12 h postactivation; 2-cell, 4-cell, and 8-cell; morula; ICM and TE of day 3.5 blastocysts. An ES cell line (R1, male) and cumulus cells were also harvested for the Hi-C experiment. For the one-cell and cleavage-stage embryos, the zona pellucidae of the embryos were removed with 0.5% pronase E (Sigma), and the embryos were then incubated in calcium-free CZB for 5 min. Polar bodies were removed by gently pipetting using a firepolished glass needle with an inner diameter of 120 μm. For morula, ICM and TE isolation, the zona pellucidae of blastocysts were removed with 0.5% pronase E. The embryos were then incubated in calcium-free CZB for 20 min, and the tight junctions of morula, TE and ICM cells were separated by gently pipetting using a pipette with an inner diameter of 40–60 μm.

### Cell culture

R1 ES cells were purchased from American Type Culture Collection (ATCC) and cultured on mitomycin C-treated MEFs in ES medium containing DMEM (Merck Millipore) supplemented with 15% (v/v) fetal bovine serum (HyClone), 1-mM l-glutamine (Merck Millipore), 0.1 mM mercaptoethanol (Merck Millipore), 1% nonessential amino acid stock (Merck Millipore), penicillin/streptomycin (100×, Merck Millipore), nucleosides (100×, Merck Millipore) and 1000 U ml^−1^ LIF (Merck Millipore). These cells tested negative for mycoplasma contamination.

### Hi-C library generation and sequencing

The generation of Hi-C libraries with a low number of cells was optimized according to a previous protocol^17^. For Hi-C library generation and sequencing, 100–500 cells were used per reaction, and at least two replicates were analyzed for each stage. All isolated cells were washed three times with a 0.5% bovine serum albumin in phosphate-buffered saline (BSA-PBS, Sigma) solution to avoid potential contamination. Briefly, SCNT embryos or mouse ESCs were cross-linked with 1% formaldehyde at room temperature (RT) for 10 min with rotation, and 2.5 M glycine was added to a final concentration of 0.2 M to quench the reaction. The mixture was incubated at RT for 10 min with rotation. A mouth pipette was used to transfer embryos or cells from a 0.5% BSA-PBS droplet into lysis buffer (10 mM Tris-HCl pH 7.4, 10 mM NaCl, 0.1 mM EDTA, 0.5% NP-40 and proteinase inhibitor cocktails (50× PIC)) under a stereoscope. Cells were lysed in 50 μl of lysis buffer on ice for at least 50 min and centrifuged at 1500 rpm for 5 min at 4 °C. The supernatant was discarded. The pellet was gently resuspended in 10 μl of 0.5% sodium dodecyl sulfate (SDS) and incubated at 62 °C for 10 min. After heating, 5 μl of 10% Triton X-100 (Sigma, 93443) was added to quench the SDS. The sample was mixed well, and excessive foaming was avoided. The sample was incubated at 37 °C for 30 min, and 5 μl of 10× NEBuffer 2 and 50 U of the MboI restriction enzyme (NEB, R0147) were added to digest the chromatin overnight or for at least 2 h at 37 °C with rotation. The sample was then incubated at 62 °C for 20 min to inactivate MboI and then cooled to RT. To fill the DNA with biotin, 3.75 μl of 0.4 mM biotin-14-dATP, 1.5 μl of 1 mM dCTP, 1.5 μl of 1 mM dGTP, 1.5 μl of 1 mM dTTP, and 10 U Klenow were added to the solution, and the reaction was carried out at 37 °C for 1.5 h with rotation. Next, 60 μl of ligation mix (38.8 μl of water, 12 μl of 10× NEB T4 DNA ligase buffer, 7 μl of 10% Triton X-100, 1.2 μl of 10 mg ml^−1^ bovine serum albumin, 1 μl of 400 U ul^−1^ T4 DNA ligase) was added. The mixture was inverted and incubated at RT for 6 hours with slow rotation. Proteins were degraded by the addition of 5 μl of 20 mg ml^−1^ proteinase K and 12 μl of 10% SDS and incubation at 55 °C for 30 min. Next, 13 μl of 5 M sodium chloride was added, and the sample was incubated at 68 °C overnight or for at least 1.5 h. DNA purification was performed by adding 1 μl of glycogen, 0.1× volume (15 μl) of 3 M sodium acetate (pH 5.2), and 1× volume (150 μl) of pure isopropanol to each tube. The mixture was inverted and incubated at −80 °C for 15 min ~ 2 h. The sample was then centrifuged at 13,000 rpm for 15 min at 4 °C. The tubes were kept on ice after centrifugation, and the supernatant was carefully removed by pipetting. All the supernatant was removed, and the pellet was washed twice with 800 μl of 75% ethanol and dissolved in 50 μl of 1× Tris buffer (10 mM Tris-HCl, pH 8, elution buffer). DNA was sheared to 300–500 bp with the Covaris S220 instrument. Biotin-labeled DNA was then pulled down with 10 μl of Dynabeads MyOne Streptavidin T1 (Life Technologies, SA-T1). The sequencing library was prepared using beads with the KAPA hyper kit. In total, 14~16 cycles of PCR amplification were performed. DNA was removed from the SA-T1 beads by heating at 98 °C for 10 min with an additional 15 μl of elution buffer. Finally, size selection was performed using AMPure XP beads. Fragments ranging in size from 200 to 500 bp were selected. All libraries were sequenced on the Illumina HiSeq X Ten or Nova platform.

### ULI-NChIP library generation and sequencing

The ULI-NChIP libraries for CCs were generated according to a previous protocol^50^. At least 500 cells were used per reaction, and two replicates were performed. All isolated cells were washed three times with a 0.5% BSA-PBS solution to avoid potential contamination. One microgram of the histone H3K9me3 antibody (39161, Active Motif) was used for each immunoprecipitation reaction. First, antibodies were bound to protein A-coated magnetic beads for at least 2 hours at 4 °C with rotation. Second, cells were seeded in 20 μl of nucleus extraction buffer (10 mM Tris-HCl (pH 8.5), 140 mM NaCl, 5 mM MgCl_2_, 0.6% NP-40, 1 mM PMSF, PIC), and MNase master mix (1× MNase master buffer, 2 mM DTT, 5% PEG6000, 30 U MNase) was then added to each sample. The sample was mixed well by gentle vortexing and then incubated at 25 °C for 7 min. Next, 5.5 μl of 100 mM EDTA was added to stop the reaction, and 5.5 μl of nuclear break buffer (1% Triton, 1% DOC solution) was added to the tube and mixed well. The samples at this stage represented sheared chromatin and were ready to be subjected to ChIP. Third, antibody-coated beads were separated with a magnetic rack, 100 μl of diluted sheared chromatin was added to each tube, and 10 μl of diluted chromatin was kept as input at 4 °C. The tubes were inverted several times to ensure that the beads were resuspended, and the samples were incubated at 4 °C under constant rotation for 2 h or overnight. The samples were then washed twice with 100 μl of ice-cold low-salt buffer (20 mM Tris-HCl (pH 8.0), 0.1% SDS, 1% Triton X-100, 2 mM EDTA, 150 mM NaCl, PIC) and twice with high-salt wash buffer (20 mM Tris-HCl (pH 8.0), 0.1% SDS, 1% Triton X-100, 2 mM EDTA, 500 mM NaCl, PIC). The tubes were incubated at 65 °C for 2 h with rotation to separate the DNA from the beads, and 100 μl of DNA and an equal volume of phenol:chloroform:isoamyl alcohol were added to the PhaseLock tubes. The samples were mixed well by vortexing and centrifuged at 13,000 rpm for 5 min; the supernatant was then transferred to a new 1.5 ml tube. Next, 10 μl of 3 M NaAc and 1 μl of glycogen were added, followed by the addition of 250 μl of ice-cold isopropanol and sufficient mixing. The tubes were placed at −20 °C for 15 min or overnight to allow precipitation. The DNA was centrifuged at 13,000 rpm for 15 min at 4 °C, the supernatant was removed, and 800 μl of 80% EtOH was added; the tube was then left for 5 min to wash the salt. The sample was centrifuged at 13000 rpm for 5 min at 4 °C, the supernatant was discarded, and the pellet was dried for 5 min at RT. DNA elution buffer was added to dissolve the pellet. The ULI-NChIP libraries were generated using the KAPA Hyper Prep Kit according to the manufacturer’s instructions. Paired-end 150-bp sequencing was performed on the Illumina HiSeq X Ten platform.

### Immunostaining

0.5 hpi, 1 hpi, 1 hpa, 6 hpa and 8-cell SCNT embryos were fixed in 4% paraformaldehyde for 1 hour at RT, and then washed twice in 0.5% BSA-PBS for 10 min. The samples were permeabilized in 0.2% Triton X-100 for 1 h at RT, washed as above. After that, samples were incubated in primary antibody at 4 °C overnight in 0.5% BSA-PBS solution, then washed twice for 10 min at RT in 0.5% BSA-PBS. The samples were incubated with secondary conjugated antibody in 0.5% BSA-PBS at RT for 1 h, washed twice as above, stained with DAPI for 15 min at RT and used for confocal imaging. α-tublin antibody (Proteintech, 66031-2-ig) was used at a concentration of 1:200.

### RNA-seq library construction and sequencing

ICM cells and TE cells were harvested as described above. For RNA-seq library construction and sequencing, 5–10 cells were used per reaction. All isolated cells were washed three times with 0.5% BSA-PBS solution to avoid potential contamination. A single-cell RNA-seq library was amplified^51^ and generated using the KAPA Hyper Prep Kit according to the manufacturer’s instructions. Single-end 50-bp sequencing was performed on the Illumina HiSeq 2500 platform.

### *Kdm4d* mRNA in vitro transcription and injection

*Kdm4d* overexpression in SCNT embryos were performed as described in previous study^50^. In brief, in vitro transcription of *Kdm4d* was performed with the mMESSAGE mMACHINE T7 Ultra Kit (Life Technologies, Grand Island, NY, USA) according to the manufacturer’s instructions. The storage concentration of mRNA was 1100 ng μl^−1^. Enucleated MII oocytes were injected with approximately 10pl of mRNA using a Piezo-driven micromanipulator.

### RNA-seq data processing

RNA-seq reads were mapped to the mm10 reference genome after cutting adaptors using hisat2^52^ with the parameters ‘--data-cufflinks --no-discordant --no-mixed --no-unal’. The expression levels of genes were calculated using DESeq2^53^.

### Histone modification ChIP-seq data processing

Histone modification ChIP-seq reads were aligned to the mm10 reference genome after cutting adaptors using bowtie2^54^ with default parameters. The alignment files were processed and merged using SAMtools^55^. PCR duplicates were removed using Picard tools (http://broadinstitute.github.io/picard/).

### Hi-C sequence data processing

Paired-end sequencing reads were trimmed for adaptor and low-quality reads. Then, the reads were processed using HiC-Pro (v2.9.0) as described^56^. The raw contact matrices were generated for each replicate at binning resolutions of 40 kb, 100 kb, and 1 Mb. The raw contact matrices were normalized using the iterative correction and eigenvector decomposition (ICE)^57^ method to correct bias and scaled to 100 million sequences to remove the effect of sequencing depth for each sample.

### Validate reproducibility of Hi-C data

The HiCRep^58^ method was used to calculate the reproducibility score between the libraries of two replicates to validate the reproducibility of the Hi-C data. We processed the raw contact matrix at resolution of 100-kb bin, and chose span size has 5 which is a tuning parameter controlling the smoothing level.

### Heatmap of interaction frequency and visualization

The triplet sparse matrix of the ICE-normalized interaction frequency was applied to plot the heatmap using HiCPlotter (v0.8.1)^59^ with a 40-kb resolution for local views, a 100-kb resolution for a whole-chromosome view and a 1-Mb resolution for a whole-genome view, and other parameters were set to default values.

### Analysis of chromatin compartments A & B

Valid contact read pairs of samples were applied to obtain the correlation coefficient matrices and PC1 values using Homer^60^ (analyzeHiC.pl and runHiCpca.pl, v4.10). We calculated the Pearson correlation coefficients (PCCs) between the PC1 values of sample replicates to validate the reproducibility of compartments using R.

To investigate dynamic chromatin compartmentalization, we first used Homer (getDiffExpression.pl -pc1) to conduct pairwise comparison analyses of the normalized 40-kb-resolution interaction frequency matrices (adjusted p-value < 0.05 and absolute value of difference >1, X chromosome excluded). We then applied *K*-means (*K* = 10) clustering to the PC1 values of these samples. The clustered PC1 values were plotted as heatmaps.

To examine the extent of chromatin compartmental segregation across the embryonic developmental stages, we calculated two classes of interactions: (1) interactions between two sites located in the same type of compartment (i.e., A-A or B-B interactions), and (2) the remaining interactions between two sites located in different types of compartments (i.e., A–B interactions). The ratios between A–B interactions and A–A or B–B interactions of each chromosome were calculated across stages and displayed as box plots (Fig. 4b). han 2-Mb in length occur mainly within TADs and are not distal interactions.

### Histone modifications in compartments

The alignment results (BAM files) of ChIP-seq reads for histone modifications (H3K4me3 and H3K27me3) and reads for the input sample were used to calculate their signals as fragment per kilobase per million mapped reads (FPKM). The signal ratios of the histone modifications to the input sample were calculated with a 10-kb window. The results (biwig files), shown in the track view with integrative genomics viewer (IGV)^61^ (Supplementary Fig. 1f), were used to calculate the histone modification signals in compartments A and B (Supplementary Fig. 1g, h).

### TAD calling

TAD boundaries were identified by the insulation score^32,62^, which was calculated using the public script matrix2insulation.pl (https://github.com/dekkerlab/cworld-dekker ‘--is 1e6 --ids 2e5 --nt 0.25’). TADs identified by directionality index (DI) scores^16^ were calculated using a public pipeline (http://chromosome.sdsc.edu/mouse/hi-c/download.html).

### Heatmap for interaction frequencies around and within TADs

TADs in the ICM stage were used for all stages because the ICM is considered to be in a mature chromatin state. All TADs were rescaled to the same length of 1.2 Mb (~average length of TADs). The average interaction frequencies in all TADs were calculated for each stage. We further normalized the matrix of average interaction frequencies by averaging the entire matrix to ensure that all stages had an equal matrix sum. We calculated the normalized interaction frequencies in 0.6-Mb regions (i.e., half of TAD size) flanking each TAD. The normalized interaction frequencies were plotted as heatmaps.

The differential average interaction matrix of TADs between two stages was calculated by subtracting the interaction matrix of the first stage from that of the second stage. The resulting matrix was plotted as a heatmap.

### DI values around TAD boundaries

The bedGraph files of DI scores were converted to bigwig files using bedGraphToBigWig^63^. First, TAD boundaries in CCs were used for CCs, 6-hpa, 12-hpa, and early-2-cell embryos. The DI values in 1.5-Mb regions flanking TAD boundaries were calculated for each stage using computeMatrix and plotProfile, and the results were shown as line plots drawn using deepTools^64^. We also generated a random control dataset as described above. DI values were calculated for the random dataset. Profiles of DI values around TAD boundaries in CCs across early stages reveal the dissolution of CC TADs. Second, TAD boundaries in the ICM were used for early-2-cell embryos, late-2-cell embryos, 4-cell embryos, 8-cell embryos and the ICM. The DI values were calculated similarly and used to show the reestablishment of TADs during the late stages.

### Distribution of histone and genomic features around TAD boundaries

The alignment results of ChIP-seq reads for histone modifications (H3K4me3 and H3K27me3) and reads for the input sample were used to calculate histone modification signals in 500-kb regions flanking TAD boundaries using a 10-kb window. The results are shown as line plots (Supplementary Fig. 1i). Similarly, the density of repeat elements, transcription start sites (TSSs) and CpG islands were calculated with window sizes of 10 kb, 100 kb, and 1 Mb using bedtools^65^ and shown as line plots (Supplementary Fig. 1j).

### Relative TAD intensity

RTI measures the states of TADs. We calculated RTI values as described in a previous study^18^. In brief, within the neighbor rectangle range that exactly embeds a TAD, each row of the interaction matrix represents interactions at a certain genomic distance that are further classified into two groups of interactions: inside and outside of TAD, denoted as *I*_in_ and *I*_out_, respectively. The ratio of the median *I*_in_ value to the median *I*_out_ value was calculated for each row. The median of the ratios of all rows is the RTI. Larger RTI values are correlated with stronger TADs. RTI values were calculated across stages and compared to examine the TAD dynamics during the development of SCNT embryos.

### Identification of H3K9me3-marked TADs

The read coverage of H3K9me3 and the genomic input in TADs were computed using multiBamSummay with default parameters and further normalized by the TAD length and total mapped reads. TADs with H3K9me3 signals higher than the input signals were defined as H3K9me3-marked TADs. A total of 438 of 2184 TADs in CCs were marked with H3K9me3.

### Contact probability analysis

The raw contact sparse matrix with a 10-kb resolution was applied to calculate the contact probability (*P(s)*) relative to the genomic distance. We first converted the linear distance to the logarithmic distance (log10) and divided the distances into bins at an interval of 0.05. We then counted the interactions in each bin and calculated *P(s)* by dividing the number of interactions in each bin by the total number of interactions in all bins. *P(s)* was normalized such that the sum over the range of distances was 1. To eliminate the strong effect of distance on the probability, we further divided the *P(s)* values by the *P(s)~s*^−1^ values as previously described^17^.

### Identification and analysis of reprogramming-resistant regions

The FRR, PRR, and RRR during the development of SCNT embryos were defined as previously described^11^. RRRs were enriched for H3K9me3 in the donor cell genome and resisted reprogramming during SCNT embryo development.

FRRs, PRRs, and RRRs overlapping the target regions were assigned to the target regions. Overlap was determined using bedtools^65^ (intersectBed -r -e -f 0.5). Thus, we determined the numbers of FRRs, PRRs and RRRs in a given set of compartments A & B and TADs.

The insulation scores in FRRs, PRRs, and RRRs and their flanking 2-Mb regions were calculated for a given stage using computeMatrix and plotProfile and shown as line plots using deepTools^64^ in a manner similar to that used for calculating the insulation scores in TADs as described above.

To calculate the relative distance to adjacent TAD boundary of each region of FRRs, PRRs, and RRRs, as described in the previous study with some modified^66^, we firstly computed the distance of the region to the nearest boundary as Dmin, and the distance of upstream boundary to downstream boundary as Dtad, the relative distance of the region was then computed as Dmin/Dtad. We then plotted the density distribution of three types of regions over all relative distance to TAD boundary using R.

### Identification of super-enhancers

As described in a previous study^35^, the published ChIP-seq H3 lysine 27 acetylation (H3K27ac) data for 2- and 8-cell fertilization-derived embryos (GSE72784)^67^ and mouse embryonic stem cells (ENCSR000CGQ) were used to predict enhancers using the tool Homer (findPeaks, v4.10)^60^ with the following parameter settings: -style superhistone -L 0 -superWindow 20. Promoter regions were excluded. These subsets of enhancers were defined as super-enhancers (560 for 2-cell embryos, 967 for 8-cell embryos, and 640 for ESCs).

### Identification of super-enhancer–promoter contact pairs (SE–P)

First, we used 100-kb-resolution interaction matrices to identify statistically significant interaction bins for each stage using Fit-Hi-C^36^ with the following parameter settings: pass 2 spline-fitting and *q*-value < 0.01. Second, we identified SE–P interactions from the above retained significant interaction bins using bedtools^65^. In brief, the promoters were defined as ±1-kb regions of the TSS. We located the recognition site of the restriction enzyme used in Hi-C library construction within each valid contact pair. The DNA fragments between each 5′ end of contact read pairs and the restriction site were treated as the interaction regions. If the region length was shorter than 500 bp, the 500-bp region extending from the restriction site was treated as the interaction region. If 50% of one region overlapped with a super-enhancer and 50% of the other region overlapped with a promoter, the interaction pair was defined as an SE–P contact pair.

### Binless normalization of Hi-C data

We used Binless^68^ algorithm to verify our key finding. Binless normalization is a resolution-agnostic method that adapts to the quality and quantity of available data. All raw valid contact pairs were used for the analysis in Binless package with default parameters. And then, we plotted the interaction signal with Zscan4d as virtual 4C around 10–14 Mb in chromosome 7.

### Analysis of differential super-enhancer–promoter interactions

To validate if the interaction between super-enhancer and *Zscan4d* is significantly different between fertilization-derived and SCNT 2-cell embryos, a differential chromatin interactions detection (FIND^37^) method was employed to find differential chromatin interactions. The ICE normalized interaction matrices of biological replicates were grouped by sample at resolution of 100 kb as input to FIND package. Then, we got *q*-values at each bin using FIND with default parameters.

### Statistics and reproducibility

Error bars in the graphical data represent the standard error of mean (SEM). For all the box plots presented in the analysis, the hings and horizontal lines within the boxes represent the 25th, 50th, and 75th percentiles and whiskers show 1.5×the inter-quartile range. All statistical significance tests have been indicated in the corresponding figure legend, and calculated using corresponding functions in R. All sequencing experiments presented in the study were independently performed at least twice and the mapping quality information is included in Supplementary Table 1.

### Reporting summary

Further information on research design is available in the Nature Research Reporting Summary linked to this article.

## Supplementary information

Supplementary Information

Reporting Summary

## Data Availability

The data that support this study are available from the corresponding authors upon reasonable request. The H3K4me3 and H3K27me3 ChIP-seq data for the 2-cell, 8-cell and ICM stages of fertilization-derived embryo development were obtained from a previous study^50^ (GSE73952). The H3K27ac ChIP-seq data for mouse embryonic stem cells (ENCSR000CGQ), 2-cell and 8-cell fertilization-derived embryos (GSE72784) were obtained from the previous study^67^. The H3K9me3 ChIP-seq data for CCs were generated in this study. The RNA-seq data for CC, SCNT 1-cell, SCNT 2-cell, IVF 1-cell and IVF 2-cell embryos were obtained from the previous study^11^ (GSE59073). The RNA-seq data for the ICM and TE of SCNT embryos were generated in this study. The raw sequence data reported in this paper have been deposited into Gene Expression Omnibus database with accession number GSE146001 [https://www.ncbi.nlm.nih.gov/geo/query/acc.cgi?acc=GSE146001] and the Genome Sequence Archive^69^ of the Beijing Institute of Genomics (BIG) Data Center^70^ with accession number CRA001431 [https://bigd.big.ac.cn/gsa/browse/CRA001431]. The source data underlying Fig. 2a–c, 3b, 4a-c, 5a, 6b and 6d and Supplementary Figs. 1a, g–h, 2a–b, d–e, 2d, 5b, 6a–b, 7a, c, d–e and 7g are provided as Source Data files.

## Source Data

Source Data

## Code availability

Custom codes used for the analysis reported in this study are available at [https://github.com/rysterzhu/Chromatin-Structure-in-SCNT].
