# Supplementary material for: Chromatin architecture reorganization in murine somatic cell nuclear transfer embryos
Source: Nat Commun. 2020 Apr 14;11:1813. doi: 10.1038/s41467-020-15607-z (PMC7156422; doi:10.1038/s41467-020-15607-z)
Supplement: Supplementary file 1 — Supplementary Information [file 41467_2020_15607_MOESM1_ESM.pdf]

Supplementary Information:

Chromatin architecture reorganization in murine somatic cell nuclear transfer embryos

Chen et al.

Supplementary Figure 1

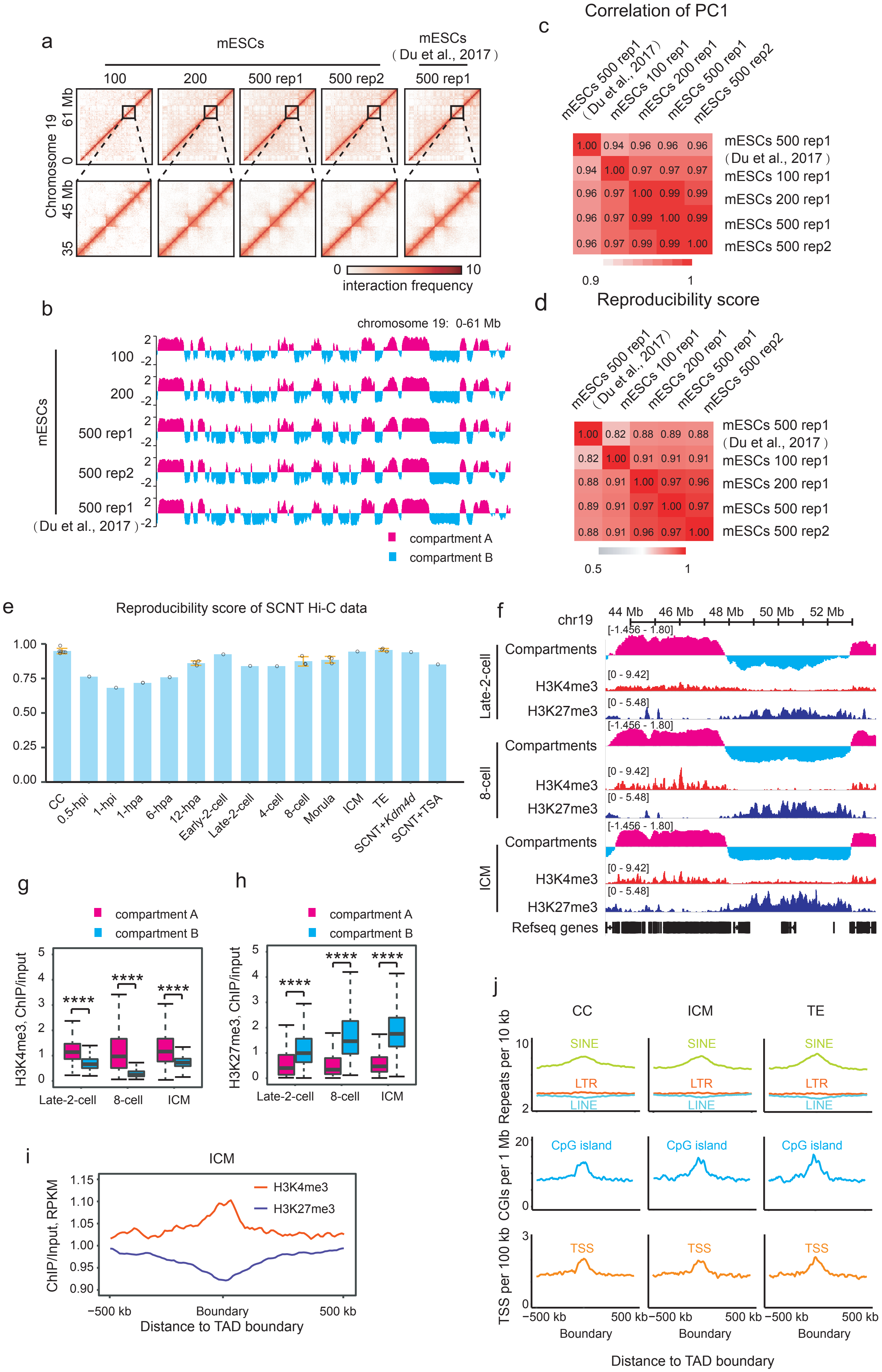

Supplementary Fig. 1 High reproducibility and validation of low-input Hi-C data. Four sets of mouse ESC low-input Hi-C data using 100, 200, and 500 cells (2 replicates from this study and one from previously published work[1]) were used for the comparison. **a**, Normalized Hi-C interaction frequencies (100-kb bin, chromosome 19) in mouse ESCs determined by low-input Hi-C. Zoomed-in views (40-kb bin) are also shown. **b**, Chromatin compartments represented by the first principal component (PC1) values in mouse ESCs calculated from the low-input Hi-C data. **c**, Heatmap showing the Pearson correlations between the PC1 values calculated from the low-input Hi-C data. Pearson correlation coefficients are shown. **d**, Heatmap showing the reproducibility score between low-input Hi-C data in this sduty and published data[1] by Hi-CRep correlation test. **e**, Reproducibility score of chromatin interaction frequencies between the replicates of Hi-C data by Hi-CRep method (CC, n = 6; 12-hpa, 8-cell, Morula, TE, n = 3). The error bars denote the SEM. Source data are provided as a Source Data file. **f**, Track view showing enrichment of H3K4me3 signals and genes in compartment A and enrichment of H3K27me3 signals in compartment B. **g-h**, H3K4me3 (**g**) and H3K27me3 (**h**) signals in genome-wide compartments A and B in late-2-cell embryos (n = 51120), 8-cell embryos (n = 51321) and the ICM (n = 51259). Boxes show 25th, 50th and 75th percentiles and whiskers show 1.5× the inter-quartile range. The two-sided p values were calculated by the Kruskal-Wallis test with Dunn's multiple comparison test and adjusted by default with the holm method (****, p-value < 0.0001). Source data and exact p-value are provided as a Source Data file. **i**, H3K4me3 and H3K27me3 signals surrounding TAD boundaries in the ICM. **j**, Distributions of repeat elements (SINE, LINE and LTR), CpG islands and TSSs (i.e., genes) around TAD boundaries in the CC, ICM and TE stages.

Supplementary Figure 2

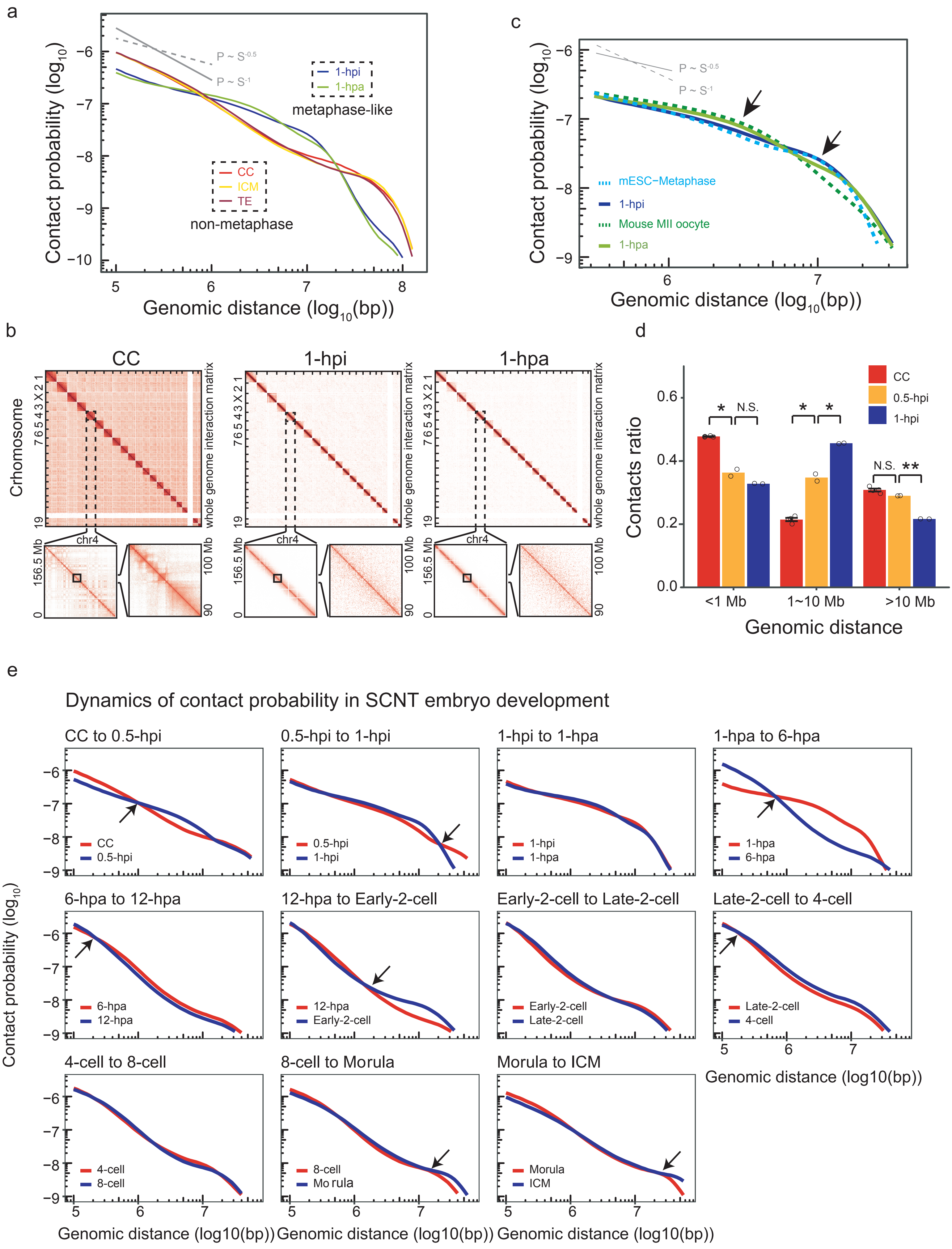

Supplementary Fig. 2 Hierarchical packaging of chromatin during the development of SCNT embryos.

a, The chromatin contact probabilities relative to genomic distance (*P(s)*) curves show two chromatin states: metaphase-like (1-hpi and 1-hpa) and nonmetaphase (CC, ICM, TE). Source data are provided as a Source Data file. **b**, Heatmaps showing whole-genome inter- and intrachromatin interaction frequencies (1-Mb bin) in CC, 1-hpi and 1-hpa zygotes. Zoomed-in views (100-kb and 40-kb bin, chromosome 4) are also shown. Source data are provided as a Source Data file. **c**, The chromatin contact probabilities relative to genomic distance (*P(s)*) curves are shown for mouse ESC metaphase chromatin, MII oocyte, 1-hpi and 1-hpa zygotes. Arrows indicate the turning points of chromatin interactions on the *P(s)* curves. The $P(s) \sim s^{-0.5}$ and $\sim s^{-1}$ curves represent the predicted mitotic and fractal globule states, respectively. **d**, Proportions of intrachromosomal interactions categorized by genomic distance (CC, n = 4; 0.5-hpi, 1-hpi, n = 2). The error bars denote SEM between replicates (*, p-value < 0.05; **, p-value < 0.01; N.S., not significant; one-sided Student's t-test). Source data and exact p-value are provided as a Source Data file. **e**, Curve plots showing chromatin interaction probabilities (*P(s)*) relative to genomic distance for every two consecutive stages. The arrows indicate the intersections on the *P(s)* curves where chromatin interactions at given genomic distances change abruptly between consecutive stages. Source data are provided as a Source Data file.

Supplementary Figure 3

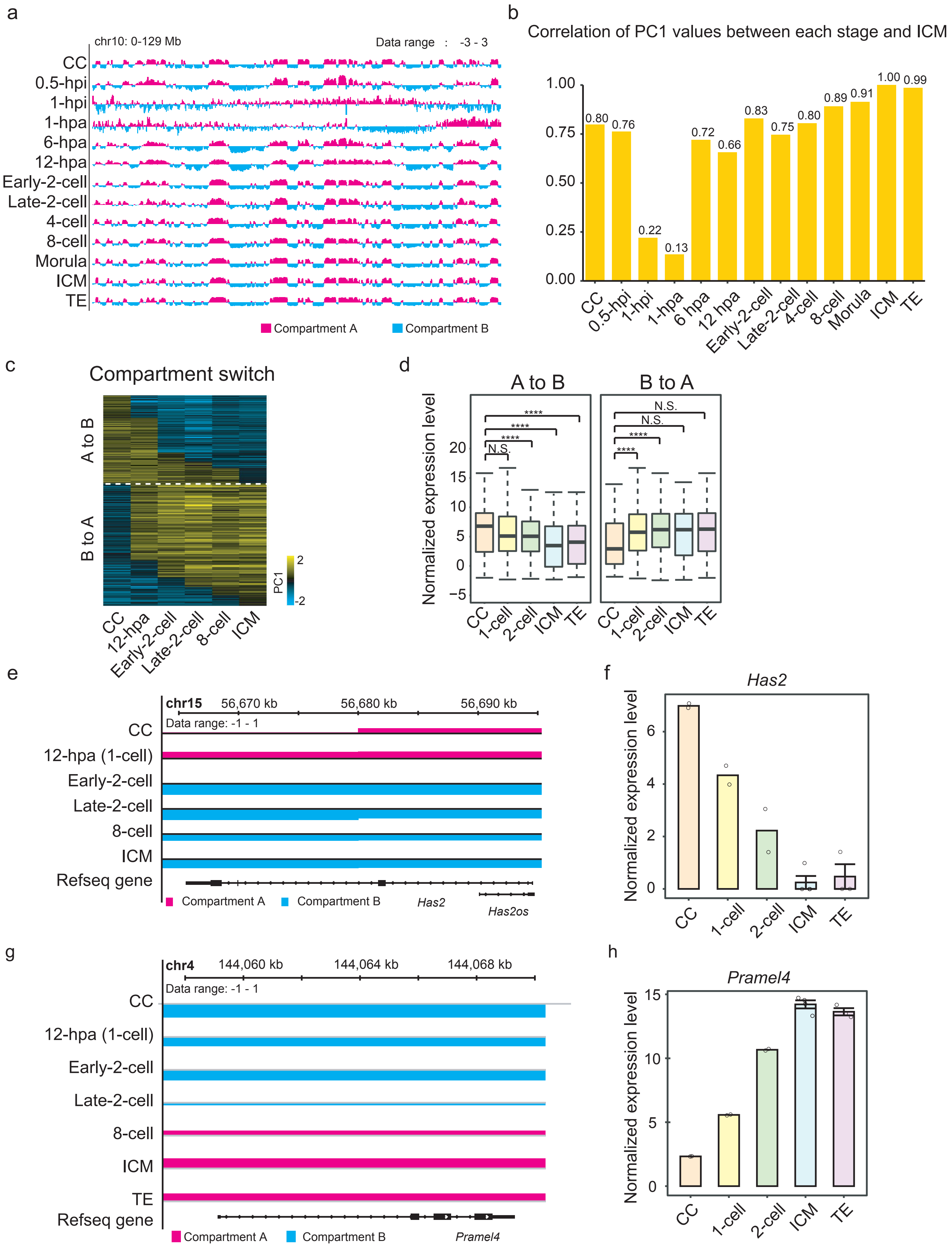

Supplementary Fig. 3 Dynamic reorganization of chromatin compartments during SCNT embryo development.

a, Chromatin compartments represented by first principal component (PC1) values across all stages. **b**, Pearson correlation of PC1 values between each stage and the ICM stage of SCNT embryos. **c**, Heatmap showing two classes of compartments. “A to B” compartments start with the “A” type and change to the “B” type during the development of SCNT embryos. “B to A” compartments exhibit the opposite trend. **d**, Gene expression levels in the “A to B” (n = 744) and “B to A” (n = 1014) compartments across the developmental stages. Boxes show 25th, 50th and 75th percentiles and whiskers show 1.5× the inter-quartile range. The two-sided p values were calculated by the Kruskal-Wallis test with Dunn’s multiple comparison test and adjusted by default with the holm method (****, p-value < 0.0001; N.S., not significant) (Supplementary Table 2). Source data and exact p-value are provided as a Source Data file. **e**, An example gene, *Has2*, within “A to B” compartments. **f**, Expression levels of *Has2* across the developmental stages of SCNT embryos (CC, 1-cell, 2-cell, n = 2; ICM, n = 4; TE, n = 3). The error bars denote SEM for replicates. **g**, An example gene, *Pramel4*, within “B to A” compartments. **h**, Expression levels of *Pramel4* across the developmental stages of SCNT embryos (CC, 1-cell, 2-cell, n = 2; ICM, n = 4; TE, n = 3). The error bars denote SEM for replicates.

Supplementary Figure 4

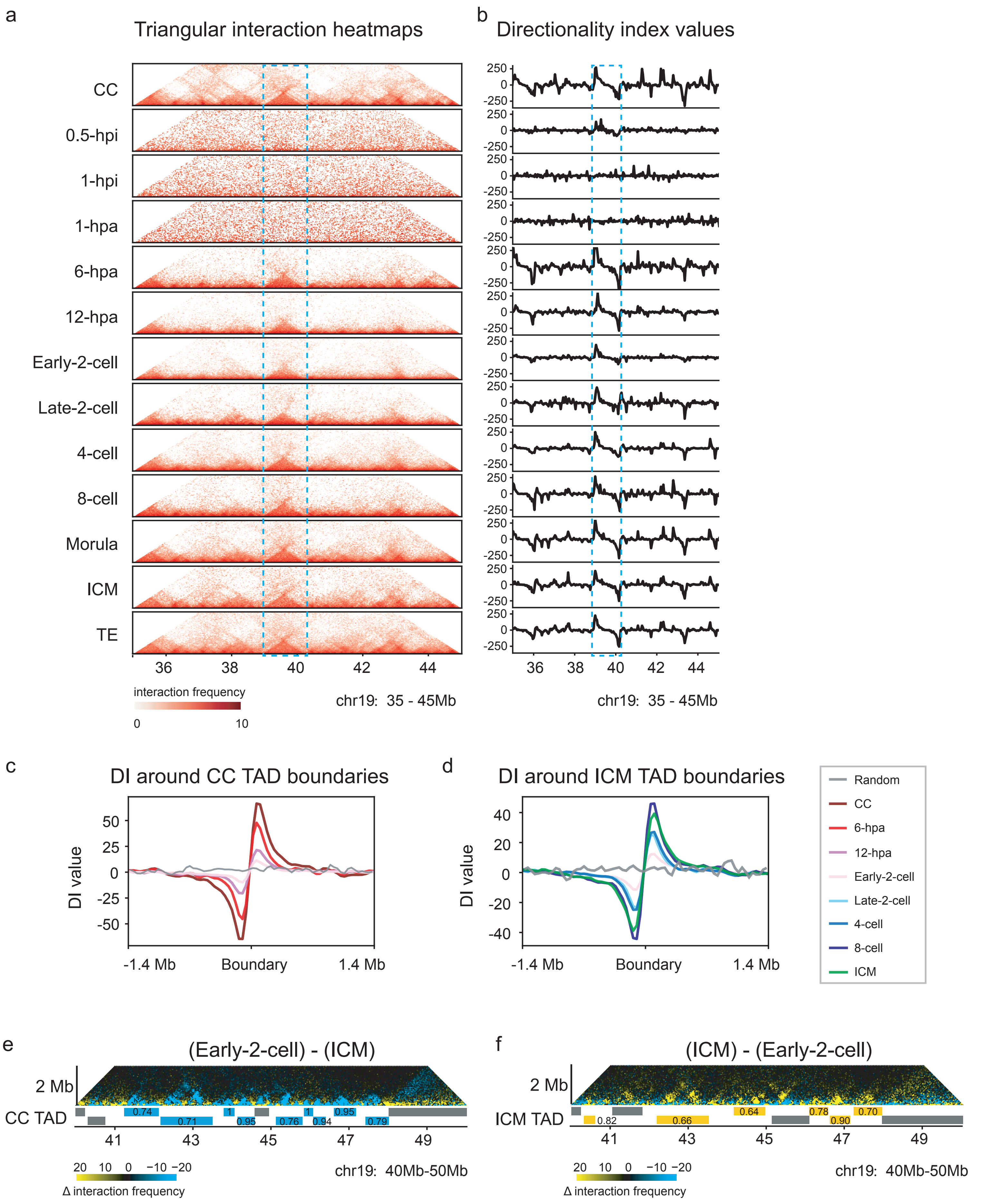

Supplementary Fig. 4 Disassembly and reestablishment of TADs during the development of SCNT embryos.

a, Heatmaps (40-kb bin) showing the dynamics of local interactions and TAD formation across all stages. The dotted box indicates a TAD. **b**, The directionality index for the corresponding region in (**a**). The dotted box indicates the TAD marked in (**a**). **c**, The average DI scores around CC TAD boundaries for CCs and early stages. DI scores generated by a random valid pair data set are also shown as a control (see Methods). **d**, Average DI scores around ICM TAD boundaries across different stages after the early-2-cell stage. **e-f**, Changes in interaction frequencies from CCs to early-2-cell embryos (**e**) and from early-2-cell embryos to the ICM (**f**). Regions with decreased and increased interaction frequencies in the later stage are shown in blue and yellow, respectively. The identified TADs with concerted increasing and decreasing interaction frequencies from early to late stages are labeled in yellow and blue, respectively. The numbers indicate the fraction of TADs with increased and decreased interaction frequencies. Other TADs without a concerted change are labeled in gray.

Supplementary Figure 5

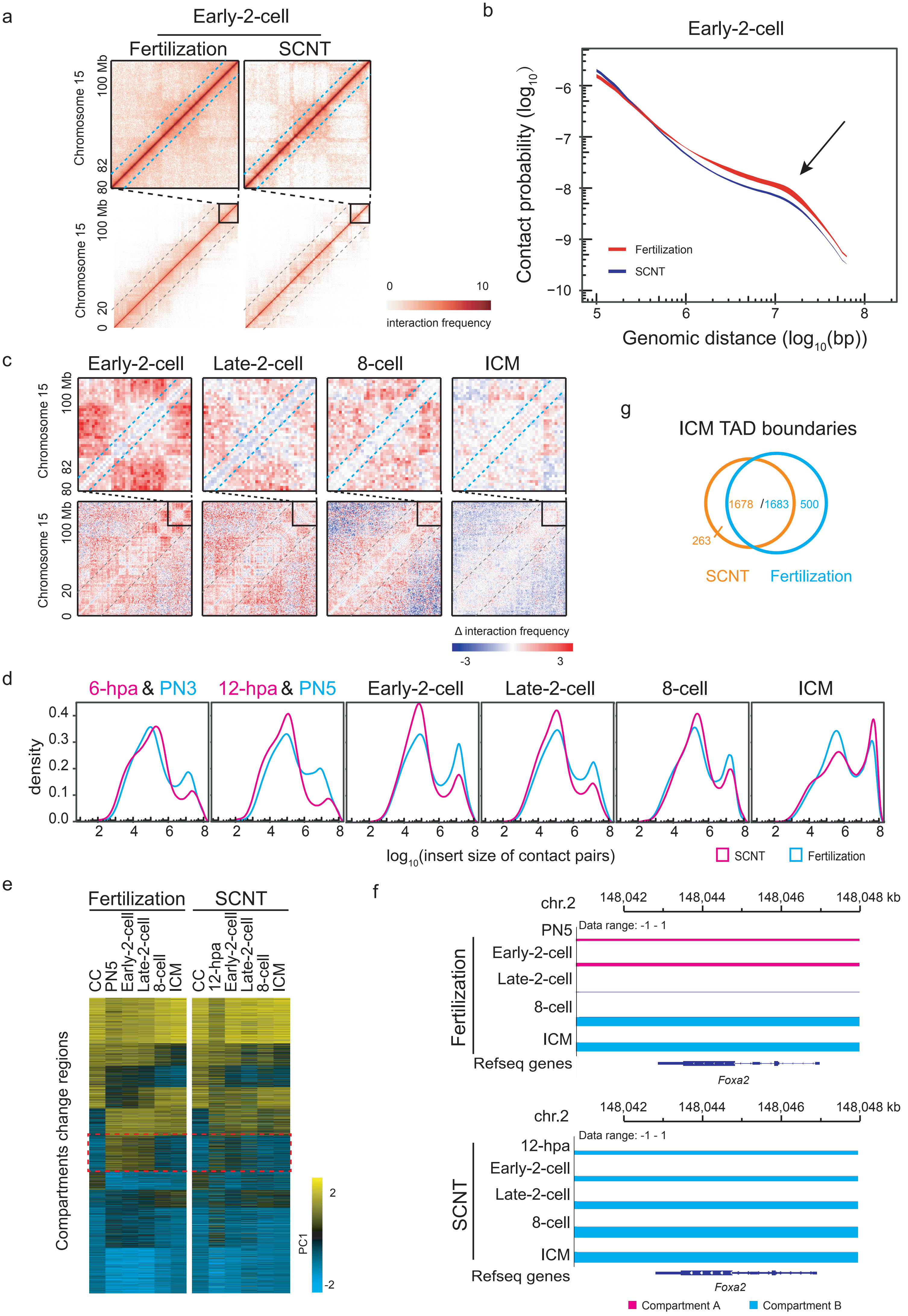

Supplementary Fig. 5 Differential chromatin organization pattern between SCNT and fertilization-derived embryos.

a, The normalized Hi-C interaction frequencies (100-kb bin, chromosome 15) in SCNT and fertilization-derived early 2-cell embryos. Zoomed-in views (40-kb bin) are also shown. The gray dashed line shows the 20 Mb distance position. The blue dashed line shows the 2 Mb distance position. **b**, The chromatin contact probabilities (*P(s)*) relative to genomic distance for SCNT and fertilization-derived early 2-cell embryos. The *P(s)* ribbon is bounded by minimum and maximum (*P(s)*) calculated from all independent replicates of Hi-C data sets. Source data are provided as a Source Data file. **c**, The differential interaction matrix (500-kb bin, chromosome 15) between fertilization-derived and SCNT in early-2-cell, late-2-cell, 8-cell embryos and ICM. Zoomed-in region within 20 Mb position was shown as well. The gray dashed line represents 20 Mb distance position. The blue dashed line shows the 2 Mb distance position. **d**, Density plot of interaction read pairs against insert size (i.e., genomic distance) across all developmental stages of SNCT and fertilization-derived embryos. **e**, The left heatmap shows genomic regions (40-kb bin) clustered by PC1 values across developmental stages of fertilization-derived embryos. The right heatmap shows PC1 values in genomic regions in the same order as those in the left heatmap across developmental stages of SCNT embryos. **f**, Track showing the compartmental state around the *Foxa2* locus across the developmental stages of fertilization-derived and SCNT embryos. *Foxa2* is located in the regions marked by the dashed box in (**e**). **g**, Overlap of TAD boundaries between SCNT and fertilization-derived ICMs.

Supplementary Figure 6

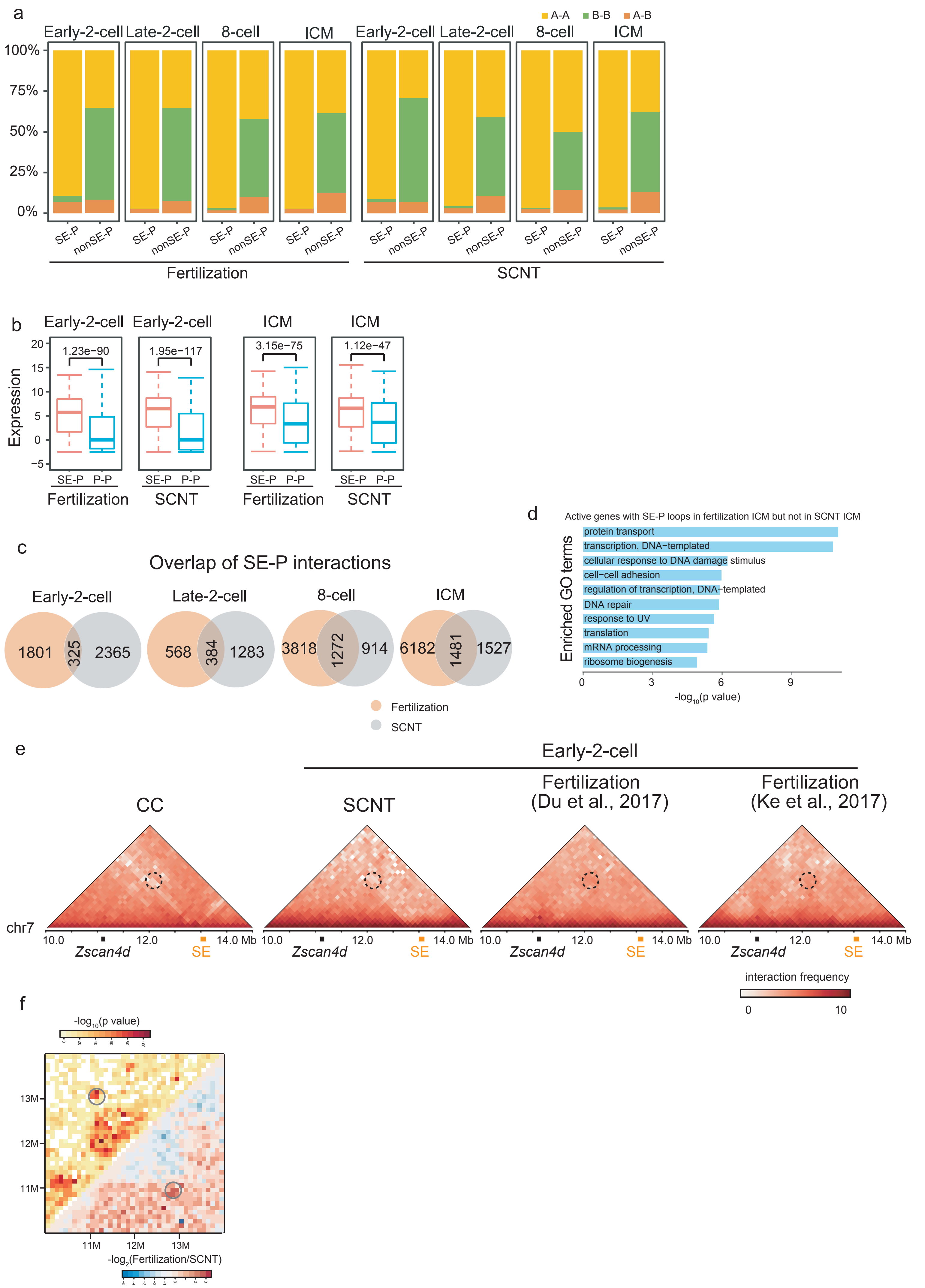

Supplementary Fig. 6 Profiling of interactions between super-enhancers and promoters in the development of SCNT and fertilization-derived embryos.

a, Stacking bar graph showing the proportions of super-enhancer ~ promoter (SE-P) interactions and nonSE-P interactions within the three types of interactions among compartments A-A, B-B and A-B across the developmental stages of SCNT and fertilization-derived embryos. Source data are provided as a Source Data file. **b**, Box plots showing the expression levels of genes associated with SE-P and promoter ~ promoter (P-P) interactions in early-2-cell embryos (Fertilization P-P, n = 1759, SE-P, n = 818; SCNT P-P, n = 1483, SE-P, n = 1160) and the ICM (Fertilization P-P, n = 8247, SE-P, n = 1286; SCNT P-P, n = 3804, SE-P, n = 1474). Boxes show 25th, 50th and 75th percentiles and whiskers show 1.5× the inter-quartile range. Statistical significance was determined by the Wilcoxon rank-sum test with two-sided. Source data are provided as a Source Data file. **c**, Overlap of SE-P interactions in early-2-cell, late-2-cell, 8-cell embryos and the ICM between SCNT and fertilization-derived embryos. **d**, GO terms for the active genes with SE-P loops in the fertilization-derived ICM but not in the SCNT ICM are enriched. **e**, Normalized Hi-C interaction frequencies (100-kb bin, chromosome 7) showing the absence of the SE-P interaction at the *Zscan4d* locus in CCs and SCNT early-2-cell embryos and the presence of this interaction in fertilization-derived embryos. **f**, Heatmap showing the differential interaction frequencies around *Zscan4d* locus between fertilization derived and SCNT early 2-cell embryos, the q-value of fold-change which are calculated using FIND are also shown. The circle indicates the SE-P interaction at *Zscan4d* locus.

Supplementary Figure 7

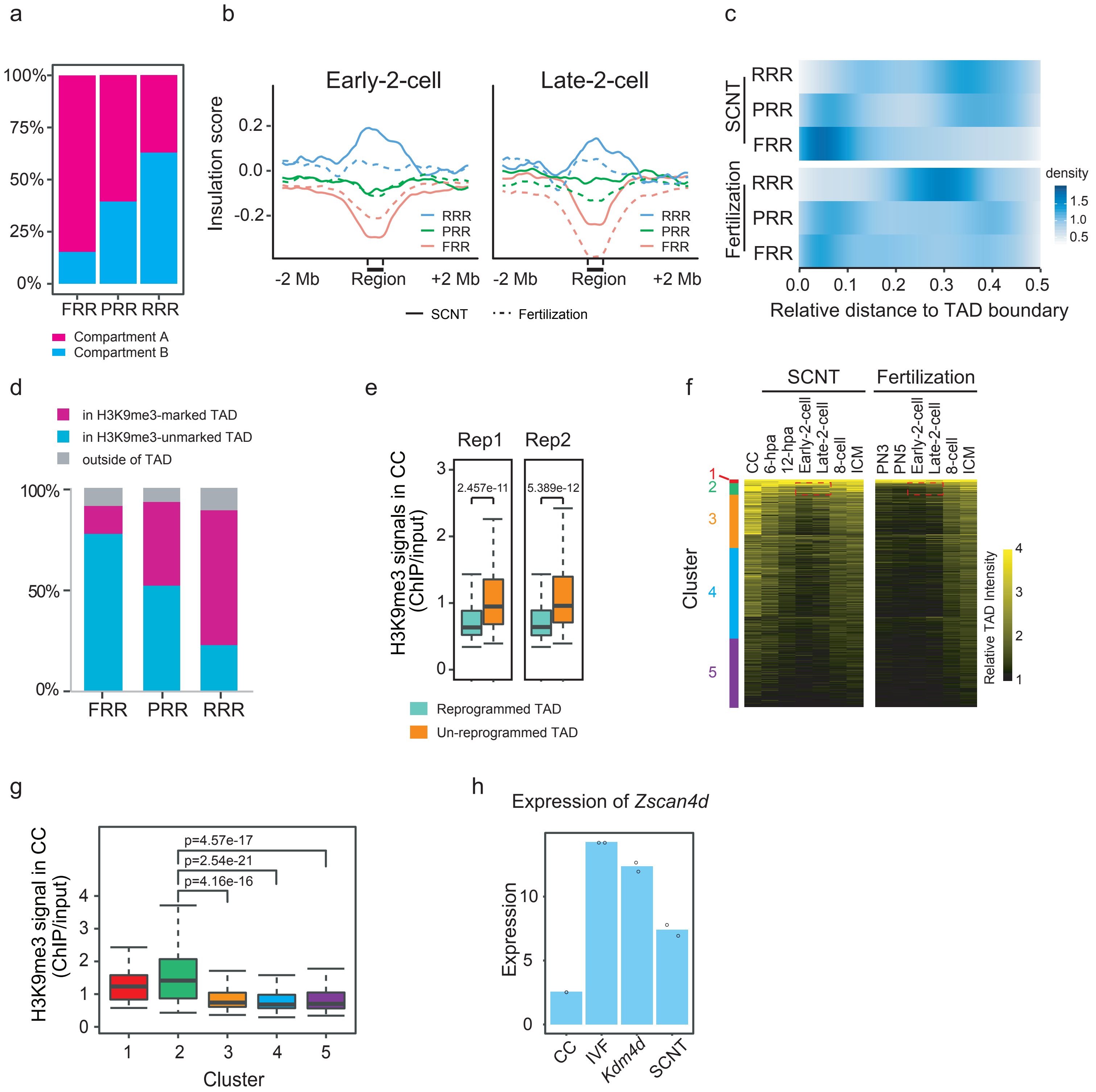

Supplementary Fig. 7 H3K9me3-enriched regions in CCs are resistant to reprogramming during mouse SCNT embryogenesis.

a, Proportions of the three types of regions (RRRs, PRRs, and FRRs, see Methods for the definitions) in compartments A and B of the SCNT ICM. Source data are provided as a Source Data file. **b**, Distributions of insulation scores around FRRs, PRRs and RRRs in early-2-cell and late-2-cell embryos. **c**, Heatmaps showing the density distribution of three types of regions over all relative distance to adjacent TAD boundary in SCNT and fertilization-derived embryos. Source data are provided as a Source Data file. **d**, Proportions of FRRs, PRRs and RRRs in H3K9me3-marked TADs, H3K9me3-unmarked TADs, and outside of TADs. TADs are SCNT CC TADs. Source data are provided as a Source Data file. **e**, H3K9me3 signal intensity in CCs of reprogrammed (n = 223) and unreprogrammed (n = 207) TADs identified by comparison of SCNT 12-hpa embryos and fertilization-derived PN5 zygotes. Boxes show 25th, 50th and 75th percentiles and whiskers show 1.5× the inter-quartile range. The two-sided p values were calculated by the Kruskal-Wallis test with Dunn's multiple comparison test and adjusted by default with the holm method. Rep1 and rep2 are two biological replicates. Source data are provided as a Source Data file. **f**, Fertilization-derived ICM TADs clustered by relative TAD intensity (RTI) across developmental stages (K-means, K = 5). The heatmaps show the RTI values in the five clusters of TADs across developmental stages of SCNT and fertilization-derived embryos. **g**, Box plot showing H3K9me3 signals in the five clusters of CC TADs in (**f**). Boxes show 25th, 50th and 75th percentiles and whiskers show 1.5× the inter-quartile range. The two-sided p values were calculated by the Kruskal-Wallis test with Dunn's multiple comparison test and adjusted by default with the holm method. Source data and exact p-value are provided as a Source Data file. **h**, Expression level of *Zscan4d* in CC, IVF, SCNT with *Kdm4d* injection and SCNT early 2-cell embryos (n = 2).

Supplementary Table 1

Sequencing information for hi-C libaries

Samples	Cell numbers	Total million pairs	Valid million pairs	Valid million pairs rmdup	million *cis*-paired reads	million *trans*-paired reads
mESC - 100	100	197.74	139.37	45.09	37.23	7.85
mESC - 200	200	229.63	162.9	92.96	76.46	16.5
mESC - 500 rep1	500	300.72	212.14	138.4	115	23.4
mESC - 500 rep2	500	224.41	158.42	96.79	81.08	15.71
Cumulus cells rep1	100	367.54	245.83	22.5	17.64	4.87
Cumulus cells rep2	100	386.28	258.25	28.94	22.57	6.37
Cumulus cells rep3	500	96.01	58.31	45.51	35.99	9.53
Cumulus cells rep4	500	95.09	59.28	40.27	32.28	7.99
0.5 hpi rep1	100	36.17	22.18	6.67	5.06	1.61
0.5 hpi rep2	200	32.26	21.01	7.15	5.72	1.43
1 hpi rep1	100	74.38	43.6	8.14	7.51	0.63
1 hpi rep2	100	26.51	16.11	3.18	2.06	1.11
1 hpa rep1	100	87.25	50.42	4.9	4.42	0.48
1 hpa rep2	100	33.52	21.81	4.03	3.52	0.51
6 hpa rep2	500	449.56	245.53	53.92	41.28	12.64
6 hpa rep3	300	480.21	207.38	59.57	47.25	12.33
12 hpa rep1	500	324.7	181.88	51.37	33.53	17.84
12 hpa rep2	500	350.76	198.72	72.4	60.38	12.02
12 hpa rep3	500	182.77	96.16	36	24.23	11.77
Early-2-cell rep3	600	241.8	152.16	87.14	66.2	20.94
Early-2-cell rep4	420	467.34	242.35	87.81	64.31	23.5
Late-2-cell rep1	200	65.17	42.7	13.94	9.6	4.35
Late-2-cell rep2	700	246.1	150.79	76.03	62.59	13.44
4-cell rep1	400	76.6	50.4	23.43	18.47	4.97
4-cell rep2	600	369.86	237.89	76.79	45.87	30.91
8-cell rep1	200	31.59	21.3	7.59	5.86	1.72
8-cell rep2	200	260.26	179.43	20.96	16.54	4.42
8-cell rep3	200	239.89	164.06	22.66	18.13	4.53
Morula rep1	100	235.39	158.73	24.91	21.21	3.7
Morula rep2	100	31.46	21.26	6.78	5.53	1.25
Morula rep3	600	338.72	216.1	55.68	40.87	14.81
ICM rep1	200	420.55	265.75	50.24	38.02	12.23
ICM rep2	300	402.67	257.9	72.92	55.35	17.58
TE rep1	500	271.15	172.28	66.8	53.31	13.49
TE rep2	400	87.62	53.44	27.66	21.32	6.34
TE rep3	500	237.8	153.88	71.37	55.61	15.76
Kdm4d early-2-cell rep1	420	487.77	268.05	96.63	70.89	25.74
Kdm4d early-2-cell rep2	420	474.42	242.33	114.21	83.77	30.43
TSA early-2-cell rep1	320	488.29	305.61	77.78	52.28	25.51
TSA early-2-cell rep2	320	486.93	296.57	36.85	24.60	12.26
