# Supplementary material for: Chromatin architecture reorganization in murine somatic cell nuclear transfer embryos
Source: Nat Commun. 2020 Apr 14;11:1813. doi: 10.1038/s41467-020-15607-z (PMC7156422; doi:10.1038/s41467-020-15607-z)
Supplement: Supplementary file 2 — Reporting Summary [file 41467_2020_15607_MOESM2_ESM.pdf]

nature research

Corresponding author(s): Shaorong Gao

Last updated by author(s): Mar 3, 2020

Reporting Summary

Nature Research wishes to improve the reproducibility of the work that we publish. This form provides structure for consistency and transparency in reporting. For further information on Nature Research policies, see Authors & Referees and the Editorial Policy Checklist .

Statistics

For all statistical analyses, confirm that the following items are present in the figure legend, table legend, main text, or Methods section.

n/a	Confirmed	
☐	☒	The exact sample size (*n*) for each experimental group/condition, given as a discrete number and unit of measurement
☐	☒	A statement on whether measurements were taken from distinct samples or whether the same sample was measured repeatedly
☐	☒	The statistical test(s) used AND whether they are one- or two-sided<br>*Only common tests should be described solely by name; describe more complex techniques in the Methods section.*
☐	☒	A description of all covariates tested
☐	☒	A description of any assumptions or corrections, such as tests of normality and adjustment for multiple comparisons
☐	☒	A full description of the statistical parameters including central tendency (e.g. means) or other basic estimates (e.g. regression coefficient) AND variation (e.g. standard deviation) or associated estimates of uncertainty (e.g. confidence intervals)
☐	☒	For null hypothesis testing, the test statistic (e.g. *F*, *t*, *r*) with confidence intervals, effect sizes, degrees of freedom and *P* value noted<br>*Give P values as exact values whenever suitable.*
☒	☐	For Bayesian analysis, information on the choice of priors and Markov chain Monte Carlo settings
☒	☐	For hierarchical and complex designs, identification of the appropriate level for tests and full reporting of outcomes
☐	☒	Estimates of effect sizes (e.g. Cohen's *d*, Pearson's *r*), indicating how they were calculated

Our web collection on statistics for biologists contains articles on many of the points above.

Software and code

Policy information about availability of computer code

Data collection	All sequence data were collected with the Illumina HiSeq X Ten or Nova platform.
Data analysis	Paired-end sequence data was trimmed adaptor and low-quality reads using cutadapt. Then the filtered reads were processed using HiC-Pro (v2.9.0).

For manuscripts utilizing custom algorithms or software that are central to the research but not yet described in published literature, software must be made available to editors/reviewers. We strongly encourage code deposition in a community repository (e.g. GitHub). See the Nature Research guidelines for submitting code & software for further information.

Data

Policy information about availability of data

All manuscripts must include a data availability statement. This statement should provide the following information, where applicable:

- Accession codes, unique identifiers, or web links for publicly available datasets
- A list of figures that have associated raw data
- A description of any restrictions on data availability

The raw sequence data reported in this paper have been deposited into Gene Expression Omnibus database with accession number GSE146001 [https://www.ncbi.nlm.nih.gov/geo/query/acc.cgi?acc=GSE146001] and the Genome Sequence Archive of the Beijing Institute of Genomics (BIG) Data Center with accession number CRA001431 [https://bigd.big.ac.cn/gsa/browse/CRA001431], both are accessible.

Field-specific reporting

Please select the one below that is the best fit for your research. If you are not sure, read the appropriate sections before making your selection.

Life sciences study design

All studies must disclose on these points even when the disclosure is negative.

Sample size	Sample sizes were determined without statistical measures, but based on prior experience with the specific experiments and widely used sizes in relevant publications within this field of research in order to ensure that it will be appropriate for statistical analysis. See Methods for detail.
Data exclusions	No data were excluded from the analyses
Replication	All the sequencing experiments includes two or more independent biological replicates.
Randomization	No randomization was used in this study.
Blinding	Experiments execution, data collection and result analysis were usually carried out by the same person, therefore no blinding was used.

Reporting for specific materials, systems and methods

We require information from authors about some types of materials, experimental systems and methods used in many studies. Here, indicate whether each material, system or method listed is relevant to your study. If you are not sure if a list item applies to your research, read the appropriate section before selecting a response.

Materials & experimental systems

n/a	Involved in the study	
☐	☒	Antibodies
☐	☒	Eukaryotic cell lines
☒	☐	Palaeontology
☐	☒	Animals and other organisms
☒	☐	Human research participants
☒	☐	Clinical data

Methods

n/a	Involved in the study	
☐	☒	ChIP-seq
☒	☐	Flow cytometry
☒	☐	MRI-based neuroimaging

Antibodies

Antibodies used	One microgram of histone H3K9me3 antibody (39161, Active Motif) was used for each immunoprecipitation reaction.
Validation	Host: Rabbit<br>Antibody type: Polyclonal<br>Citation:<br>Jain, S. U., Do, T. J., et al. (2019), 'PFA ependymoma-associated protein EZHIP inhibits PRC2 activity through a H3 K27M-like mechanism.', Nat Commun, 10 (1), pp. 2146<br>Duempelmann, L., Mohn, F., et al. (2019), 'Inheritance of a Phenotypically Neutral Epimutation Evokes Gene Silencing in Later Generations.', Mol Cell, 74 (3), pp. 534-541.e4<br>Wu, S., Fatkhutdinov, N., et al. (2019), 'ARID1A spatially partitions interphase chromosomes.', Sci Adv, 5 (5), pp. eaaw5294

Eukaryotic cell lines

Policy information about cell lines

Cell line source(s)	The R1 ES cells were purchased from the American Type Culture Collection (ATCC).
Authentication	No further authenticated.
Mycoplasma contamination	Theses cells were tested negative for mycoplasm contamination.
Commonly misidentified lines (See ICLAC register)	None of the cell lines used in this study is listed in the database of commonly misidentified cell lines maintained by ICLAC.

Animals and other organisms

Policy information about studies involving animals; ARRIVE guidelines recommended for reporting animal research

Laboratory animals	Eight- to ten-week-old B6D2F1(C57BL/6×DBA/2) female mice

Wild animals	The study did not involve wild animals.
Field-collected samples	The study did not involve samples collected from the field.
Ethics oversight	All experiments were performed in accordance with the University of Health Guide for the Care and Use of Laboratory Animals and were approved by the Biological Research Ethics Committee of Tongji University.

Note that full information on the approval of the study protocol must also be provided in the manuscript.

ChIP-seq

Data deposition

- [x] Confirm that both raw and final processed data have been deposited in a public database such as GEO.
- [ ] Confirm that you have deposited or provided access to graph files (e.g. BED files) for the called peaks.

Data access links<br>*May remain private before publication.*	https://www.ncbi.nlm.nih.gov/geo/query/acc.cgi?acc=GSE146001 or<br>http://bigd.big.ac.cn/gsa/browse/CRA001431
Files in database submission	CC_H3K9me3_rep1.R1.fq.gz<br>CC_H3K9me3_rep1.R2.fq.gz<br>CC_H3K9me3_rep2.R1.fq.gz<br>CC_H3K9me3_rep2.R2.fq.gz
Genome browser session<br>(e.g. UCSC)	NA

Methodology

Replicates	Two replicates were adopted.
Sequencing depth	About 80 million reads pairs for each replicate.
Antibodies	As described above.
Peak calling parameters	NA
Data quality	See Supplementary Figures.
Software	Bowtie2.
